# Supplementary material for: Day-to-day variability of knee pain and the relationship with physical activity in people with knee osteoarthritis: an observational, feasibility study using consumer smartwatches
Source: BMJ Open. 2023 Mar 13;13(3):e062801. doi: 10.1136/bmjopen-2022-062801 (PMC10016308; doi:10.1136/bmjopen-2022-062801)
Supplement: Supplementary data [file bmjopen-2022-062801supp003.pdf]

BMJ Publishing Group Limited (BMJ) disclaims all liability and responsibility arising from any reliance placed on this supplemental material which has been supplied by the author(s)

R code used for analysis

```{r data, echo = TRUE}
pain <- read.csv('PainArani.csv')
koos <- read.csv('koosArani.csv')
steps <- read.csv('daily_steps.csv')
demographics <- readxl::read_excel('baseline.xlsx')
```

Remove artifacts

For the pain dataset, we decided to only include those who had pain scores >0 (as we believed that scores of 0 may have been by accident).

Pain scores of zero were treated as missing values, due to the interface of the smartwatch app occasionally generating zero-values by accident as an error in data input.

Of `r nrow(pain)` total records, `r sum(pain$pain == 0, na.rm = TRUE)` had value zero and were removed.

This may include both 'true' zeros as well as those that were entered by mistake; we have no way to tell which might be which.

```{r artifacts}
pain <- subset(pain, pain > 0)
```

Clusters

Cluster participants into three groups based on the means and variances of their morning and afternoon pain scores. We will use the *k*-means algorithm for this.

```{r clusters}
library(lubridate)
library(dplyr)
```

```
pain_summary <- pain %>%
  # Include 'overall morning' and 'overall pain' scores only.
  filter(grepl('MORNING|AFTERNOON', notification_type)) %>%
  group_by(user_id, notification_type) %>%
  # Compute summary statistics for each user.
  summarise(mean = mean(pain), var = var(pain)) %>%
  # Housekeeping
  rename(type = notification_type) %>%
  mutate(type = tolower(gsub('PAIN_OVERALL_', '', type)))

# Reshape the dataset.
pain_wide <- pain_summary %>%
  tidyr::pivot_wider(user_id, names_from = type, values_from =
c(mean, var)) %>%
  na.omit() # remove user 37 who now has no data points for
afternoon pain (after removing zeros)

knitr::kable(pain_wide %>%
               transmute(user_id,
                         mean_morning, sd_morning =
sqrt(var_morning),
                         mean_afternoon, sd_afternoon =
sqrt(var_afternoon)),
             digits = 1, caption = 'Means and standard deviations of
pain scores by user')

# Apply the k-means algorithm to the means and variances.
# Run 10 times to avoid risk of local optima.
km <- kmeans(pain_wide[, -1], 3, nstart = 10)
clusters <- tibble(user_id = pain_wide$user_id, cluster =
km$cluster)
```

We create a lookup table so it is easy to cross-reference the alpha-numeric Koalap IDs, the numeric user IDs and the cluster assignments.

```
```{r lookup}
```
```

```
user_lookup <- pain %>%
  distinct(user_id, KOALAP.Name) %>%
  left_join(clusters, by = 'user_id')
```

Baseline demographics

To calculate BMI for each participant, convert to the appropriate units, then

use the formula

\[\text{BMI}_{\text{kg m}^{-2}} = \frac{\text{Weight}_\text{kg}}{(\text{Height}_{\text{m}})^2}.\]

```{r BMI}
demographics <- subset(demographics, `Koalap ID` != '23')
demographics <- within(demographics, {
  # Fix malformatted user ID:
  `Koalap ID` <- ifelse(`Koalap ID` == '29 & 5', 5, `Koalap ID`)
  Weight_kg <- as.numeric(gsub(' kg', '', Weight))
  feet <- as.numeric(substring(Height, 1, 1))
  inches <- as.numeric(gsub('[0-9] ft ?', '', Height))
  inches[is.na(inches)] <- 0
  Height_m <- 2.54 * (12 * feet + inches) / 100
  BMI <- Weight_kg / Height_m^2
})
```

Knee injury and osteoarthritis outcome scores (Koos)

We need to convert the categorical Koos responses to numeric values and get total scores for each participant for each month.

```{r KOOS}
koos_scale <- c(None = 0, Mild = 1, Moderate = 2, Severe = 3, Extreme = 4)

# Convert categorical responses to numeric values.
```

````
koos_num <- koos %>%
  mutate_at(vars(-(1:3)), function(x) koos_scale[x]) %>%
  mutate(timestamp = as_datetime(timestamp),
         month = round_date(timestamp, 'months')) %>%
  rename(KOALAP.Name = user_id) %>%
  left_join(user_lookup, by = 'KOALAP.Name') %>%
  # Some users answered questions multiple times. Take the latest response.
  group_by(user_id, month) %>% filter(row_number() == n()) %>%
  # We are only interested in questions P2 to P9.
  select(-KOOS_P1, -starts_with('KOOS_A'))

# Calculate monthly Koos for each participant, as a percentage.
total_possible_koos <- sum(grepl('KOOS', colnames(koos_num))) * 4 # = 32
koos_totals <- koos_num %>%
  tidyr::pivot_longer(starts_with('KOOS_'), names_to = 'question') %>%
  group_by(user_id, month) %>%
  summarise(koos_score = 100 * sum(value) / total_possible_koos)

# Check the resulting scores are valid.
if (any(koos_totals$koos_score > 100))
  stop('Koos percentage cannot be more than 100')
```

Compare Koos with daily pain trajectories.

```{r koos v pain, fig.width = 10, fig.height = 7}
koos_totals_numeric_ids <- koos_totals %>%
  ungroup() %>%
  left_join(user_lookup, by = 'user_id') %>%
  mutate(month = as.Date(month)) %>%
  filter(!is.na(user_id), user_id != 23)

pain %>%
````

````
  mutate(timestamp.Day = as_date(timestamp.Day)) %>%
  filter(notification_type.Number == 15, # morning pain only
         !is.na(user_id), user_id != 23) %>% # get rid of user whose id is 'NA'
  ggplot() +
  aes(timestamp.Day, pain) +
  geom_line() +                  # daily pain scores
  geom_point(aes(month, koos_score / 10), data = koos_totals_numeric_ids,
             colour = 'red', size = 2) +
  facet_wrap(~as.numeric(as.factor(user_id))) +
  scale_y_continuous(breaks = seq(0, 10, by = 5)) +
  labs(x = 'Date', y = 'Pain / KOOS',
       title = 'Monthly KOOS scores and daily pain scores',
       subtitle = 'Based on overall morning pain scores')

pain %>%
  mutate(timestamp.Day = as_date(timestamp.Day)) %>%
  filter(notification_type.Number == 14, # afternoon pain only
         !is.na(user_id), user_id != 23) %>% # get rid of user whose id is 'NA'
  ggplot() +
  aes(timestamp.Day, pain) +
  geom_line() +                  # daily pain scores
  geom_point(aes(month, koos_score / 10), data = koos_totals_numeric_ids,
             colour = 'red', size = 2) +
  facet_wrap(~as.numeric(as.factor(user_id))) +
  scale_y_continuous(breaks = seq(0, 10, by = 5)) +
  labs(x = 'Date', y = 'Pain / KOOS',
       title = 'Monthly KOOS scores and daily pain scores',
       subtitle = 'Based on overall afternoon pain scores')
```
````

Step counts
````

Calculate the mean and variance of step counts per user, and link these data with the clustering assignments.

````
```{r steps}
steps_summary <- steps %>%
  rename(KOALAP.Name = user_id) %>%
  left_join(user_lookup, by = 'KOALAP.Name') %>%
  group_by(user_id = user_id) %>%
  summarise(mean = mean(steps), var = var(steps), sd = sqrt(var))
```

```{r step trajectories, fig.width = 10, fig.height = 5}
steps %>%
  rename(KOALAP.Name = user_id) %>%
  left_join(user_lookup, by = 'KOALAP.Name') %>%
  # Get rid of user 'NA' and user 23 who disappeared.
  filter(!is.na(user_id), user_id != 23) %>%
  ggplot() +
  aes(as.Date(date), steps, group = user_id) +
  geom_point(size = 1) +
  facet_wrap(~as.numeric(as.factor(user_id))) +
  scale_x_date(date_breaks = '1 month', date_labels = '%b') +
  scale_y_continuous(labels = function(x) ifelse(x == 0, '0',
scales::unit_format(1, scale = 1/1000, unit = 'k', sep = '')(x))) +
  labs(x = NULL, y = 'step count') #+
  #theme(strip.text = element_blank())
```

```{r}
steps_summary %>%
  left_join(user_lookup) %>%
  group_by(cluster) %>%
  summarise(mean = mean(mean),
            sd = sqrt(mean(var)),
            var = mean(var))
````
````

````
steps_summary %>%
  left_join(user_lookup2) %>%
  group_by(cluster) %>%
  summarise(mean = mean(mean),
            sd = sqrt(mean(var)),
            var = mean(var))
```


## Demographics by cluster


Let's plot step counts and body mass index (BMI).


```{r step counts by BMI}
steps %>%
  rename(KOALAP.Name = user_id) %>%
  left_join(user_lookup, by = 'KOALAP.Name') %>%
  # Summary statistics of step counts.
  group_by(user_id) %>%
  summarise(Q1 = quantile(steps, .25),
            Q3 = quantile(steps, .75),
            median = median(steps),
            min = min(steps), max = max(steps)) %>%
  # Housekeeping (match up the data types).
  mutate(user_id = as.character(user_id)) %>%
  # Join with BMI data.
  left_join(demographics, by = c(user_id = 'Koalap ID')) %>%
  filter(!is.na(user_id), user_id != '23') %>%
  ggplot() + aes(BMI, median) +
  geom_linerange(aes(ymin = min, ymax = max), alpha = .3) + # min to max
  geom_linerange(aes(ymin = Q1, ymax = Q3), linetype = 'solid') + # IQR
  geom_point() +
  theme_classic() +
  ylab('Step counts')
```
````
````

````
### BMI distribution

```{r BMI qq}
qqnorm(demographics$BMI, main = 'Normal quantile-quantile plot of
BMI',
       col = c(M = 'steelblue', F = 'tomato2')[demographics$Gender],
       pch = c(M = 16, F = 17)[demographics$Gender])
qqline(demographics$BMI)
legend('bottomright',
       legend = c('M', 'F'),
       col = c('steelblue', 'tomato2'),
       pch = 16:17)
```

In tabular form.

```{r BMI table}
summary(demographics$BMI) %>%
  as.list %>%
  as_tibble %>%
  knitr::kable()
```

Standard deviation:

```{r}
sd(demographics$BMI)
```

### Age distribution

```{r age qq}
qqnorm(demographics$Age, main = 'Normal quantile-quantile plot of
age',
       col = c(M = 'steelblue', F = 'tomato2')[demographics$Gender],
````
````

````
       pch = c(M = 16, F = 17)[demographics$Gender])
qqline(demographics$Age)
legend('bottomright', legend = c('M', 'F'),
       col = c('steelblue', 'tomato2'),
       pch = c(16, 17))
```

```{r}
summary(demographics$Age) %>%
  as.list %>%
  as_tibble %>%
  knitr::kable()
```

```{r}
sd(demographics$Age)
```

## Quality of life score

```{r qol}
pain %>%
  filter(notification_type == 'PAIN_QUALITY_OF_LIFE',
         !user_id %in% c(23, 37)) %>%
  group_by(user_id) %>%
  summarise(mean_qol = mean(pain),
            var_qol = var(pain)) %>%
  left_join(user_lookup, by = 'user_id') %>%
  group_by(cluster) %>%
  summarise(mean = mean(mean_qol),
            var = mean(var_qol, na.rm = TRUE)) %>%
  knitr::kable(col.names = c('Cluster',
                             'Mean(QoL score)',
                             'Variance(QoL score)'),
````

```
              digits = 2, caption = 'Average within-person mean and
average within-person variance (excluding participants who gave only
one QoL response)')
```

Difference in morning and afternoon pain

```{r morning afternoon difference, fig.width = 8, fig.height = 8, fig.cap = 'Jittered scatter plot of daily overall afternoon pain against overall morning pain, by participant, with a line of $y = x$'}
pain %>%
  filter(grepl('MORNING|AFTERNOON', notification_type),
         user_id != 23) %>%
  mutate(type = tolower(gsub('PAIN_OVERALL_', '', notification_type)),
         date = as.Date(timestamp.Day)) %>%
  tidyr::pivot_wider(c(user_id, date), names_from = type, values_from = pain) %>%
  tidyr::unnest() %>%
  ggplot() +
  aes(morning, afternoon) +
  geom_abline(intercept = 0, slope = 1) +
  geom_jitter(alpha = .5, width = .25, height = .25) +
  facet_wrap(~as.numeric(as.factor(user_id))) +
  scale_x_continuous('Morning pain', breaks = c(0, 5, 10)) +
  scale_y_continuous('Afternoon pain', breaks = c(0, 5, 10))
```

```{r morning afternoon timeline, fig.width = 8, fig.height = 8, fig.cap = 'Difference between morning and afternoon overall pain scores, over time, by participant'}
pain %>%
  filter(grepl('MORNING|AFTERNOON', notification_type),
         user_id != 23) %>%
  mutate(type = tolower(gsub('PAIN_OVERALL_', '', notification_type)),
         date = as.Date(timestamp.Day)) %>%
```

```
  tidyr::pivot_wider(c(user_id, date), names_from = type,
values_from = pain) %>%
  tidyr::unnest() %>%
  ggplot() + aes(date, afternoon - morning) +
  geom_line() + geom_point() +
  facet_wrap(~ as.numeric(as.factor(user_id))) +
  xlab(NULL) +
  scale_y_continuous('(afternoon pain) - (morning pain)',
                     breaks = seq(-4, 4, by = 2), minor_breaks =
NULL)
```

```{r blandaltman, fig.width = 8, fig.height = 8, fig.caption =
'Tukey mean-difference plots (aka Bland--Altman plots) for morning
and afternoon pain'}
pain %>%
  filter(grepl('MORNING|AFTERNOON', notification_type),
         user_id != 23) %>%
  mutate(type = tolower(gsub('PAIN_OVERALL_', '',
notification_type)),
         date = as.Date(timestamp.Day)) %>%
  tidyr::pivot_wider(c(user_id, date), names_from = type,
values_from = pain) %>%
  tidyr::unnest() %>%
  mutate(diff = afternoon - morning,
         avg = (afternoon + morning) / 2) -> bland_altman

bland_altman_stats <- bland_altman %>%
  #filter(!is.na(diff)) %>% # weird bug where userid 42 (plot id 25)
gets omitted here?
  group_by(user_id) %>%
  summarise(mean = mean(diff, na.rm = TRUE),
            lower = mean(diff, na.rm = TRUE) - 1.96 * sd(diff, na.rm
= TRUE),
            upper = mean(diff, na.rm = TRUE) + 1.96 * sd(diff, na.rm
= TRUE))

ggplot(bland_altman) +
  aes(avg, diff) +
```

```
  facet_wrap(~ as.integer(as.factor(user_id))) +
  scale_x_continuous('average of lunchtime and early evening pain',
                     breaks = seq(0, 10, by = 2), minor_breaks =
NULL) +
  scale_y_continuous('(early evening pain) - (lunchtime pain)',
                     breaks = seq(-5, 5, by = 5), minor_breaks =
NULL) +
  geom_hline(aes(yintercept = lower), data = bland_altman_stats,
             linetype = 'dashed') +
  geom_hline(aes(yintercept = upper), data = bland_altman_stats,
             linetype = 'dashed') +
  geom_hline(aes(yintercept = mean), data = bland_altman_stats) +
  geom_point()
```

Correlation between step count and pain

```{r correlation, fig.width = 8, fig.height = 8}
pain_and_steps <- pain %>%
  filter(notification_type == 'PAIN_OVERALL_MORNING', user_id != 23)
%>%
  transmute(user_id, date = as.Date(timestamp.Day), pain) %>%
  inner_join(steps %>%
               left_join(user_lookup, by = c(user_id =
'KOALAP.Name')) %>%
               transmute(user_id = user_id.y, steps, cluster, date =
as.Date(date)))

pain_and_steps %>%
  ggplot() + aes(pain, steps) +
  #geom_smooth(method = lm, se = FALSE) +
  geom_jitter(alpha = .5, width = .25, height = 0) +
  facet_wrap(~as.numeric(as.factor(user_id))) +
  scale_x_continuous('Lunchtime pain', breaks = c(0, 5, 10)) +
    scale_y_continuous('Steps', labels = function(x)
      ifelse(x == 0, '0', scales::unit_format(1, scale = 1/1000,
unit = 'k', sep = '')(x)))
```

```
### Redo but for afternoon pain


pain_and_steps_pm <- pain %>%
  filter(notification_type == 'PAIN_OVERALL_AFTERNOON', user_id != 23)  %>%
  transmute(user_id, date = as.Date(timestamp.Day), pain) %>%
  inner_join(steps %>%
               left_join(user_lookup, by = c(user_id = 'KOALAP.Name')) %>%
               transmute(user_id = user_id.y, steps, cluster, date = as.Date(date)))


pain_and_steps_pm %>%
  ggplot() + aes(pain, steps) +
  #geom_smooth(method = lm, se = FALSE) +
  geom_jitter(alpha = .5, width = .25, height = 0) +
  facet_wrap(~as.numeric(as.factor(user_id))) +
  scale_x_continuous('Early evening pain', breaks = c(0, 5, 10)) +
    scale_y_continuous('Steps', labels = function(x)
      ifelse(x == 0, '0', scales::unit_format(1, scale = 1/1000, unit = 'k', sep = '')(x)))
```

If we wanted to compute a cohort-level correlation we could do so via the **rmcorr** package (we don't do that, here).

```{r}
pain_and_steps %>%
  group_by(user_id) %>%
  summarise(step_pain_correlation = cor(pain, steps),
            conf_int = if (n() > 2) {
              paste(round(cor.test(pain, steps)$conf.int, 2), collapse = ',')
            } else {
              NA_character_
```

```
            }) %>%
  mutate(`ID on graphs` = as.numeric(as.factor(user_id))) %>%
  knitr::kable(col.names = c('Koalap ID', 'Correlation', 'Confidence
interval', 'User ID for graphs'),
               digits = 2, align = 'rrrr',
               caption = 'Correlation between same-day morning pain
score and step count, by user')


pain_and_steps_pm %>%
  group_by(user_id) %>%
  summarise(step_pain_correlation = cor(pain, steps),
            conf_int = if (n() > 2) {
              paste(round(cor.test(pain, steps)$conf.int, 2),
collapse = ',')
            } else {
              NA_character_
            }) %>%
  mutate(`ID on graphs` = as.numeric(as.factor(user_id))) %>%
  knitr::kable(col.names = c('Koalap ID', 'Correlation', 'Confidence
interval', 'User ID for graphs'),
               digits = 2, align = 'rrrr',
               caption = 'Correlation between same-day morning pain
score and step count, by user')
```

All of the analysis above excluded zeros from the analysis. Here we will redo it with zeros included, to highlight artifacts:

```
```{r raw scores, fig.width = 10, fig.height = 8}
pain0 <- read.csv('PainArani.csv')


pain0 %>%
  filter(grepl('MORNING', notification_type),
         user_id != 23) %>%
  mutate(date = as.Date(timestamp.Day)) %>%
  ggplot() + aes(date, pain) +
  geom_line() + geom_point(size = 1) +
```
```

```
  facet_wrap(~ as.numeric(as.factor(user_id))) +
  xlab(NULL) +
  scale_y_continuous('lunchtime pain',
                     breaks = seq(0, 10, by = 2), minor_breaks =
NULL)

pain0 %>%
  filter(grepl('AFTERNOON', notification_type),
         user_id != 23) %>%
  mutate(date = as.Date(timestamp.Day)) %>%
  ggplot() + aes(date, pain) +
  geom_line() + geom_point(size = 1) +
  facet_wrap(~ as.numeric(as.factor(user_id))) +
  xlab(NULL) +
  scale_y_continuous('early evening pain',
                     breaks = seq(0, 10, by = 2), minor_breaks =
NULL)

pain0 %>% count(user_id, pain) %>% filter(pain == 0)
```

```{r}
pain0 %>%
  filter(grepl('MORNING', notification_type),
         user_id == 18) %>%
  mutate(date = as.Date(timestamp.Day)) %>%
  ggplot() + aes(date, pain) +
  geom_line() + geom_point(size = 1) +
  xlab(NULL) +
  scale_y_continuous('lunchtime pain',
                     breaks = seq(0, 10, by = 2), minor_breaks =
NULL,
                     limits = c(0, 10))
```
