# Supplementary material for: Day-to-day variability of knee pain and the relationship with physical activity in people with knee osteoarthritis: an observational, feasibility study using consumer smartwatches
Source: BMJ Open. 2023 Mar 13;13(3):e062801. doi: 10.1136/bmjopen-2022-062801 (PMC10016308; doi:10.1136/bmjopen-2022-062801)
Supplement: Supplementary data [file bmjopen-2022-062801supp001.pdf]

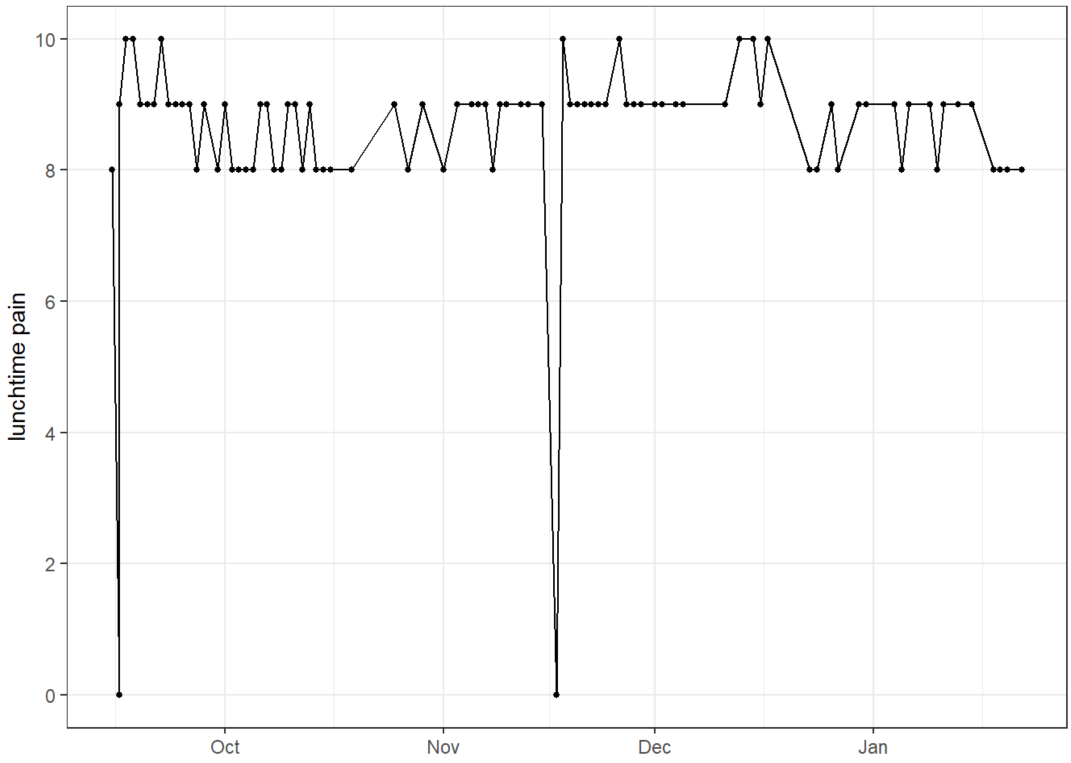

Figure A1: An example of a user with what we believe shows 'false zeros' (i.e., entered by mistake due to user interface error)
