# Supplementary figures and images for: Day-to-day variability of knee pain and the relationship with physical activity in people with knee osteoarthritis: an observational, feasibility study using consumer smartwatches
Source: BMJ Open. 2023 Mar 13;13(3):e062801. doi: 10.1136/bmjopen-2022-062801 (PMC10016308; doi:10.1136/bmjopen-2022-062801)

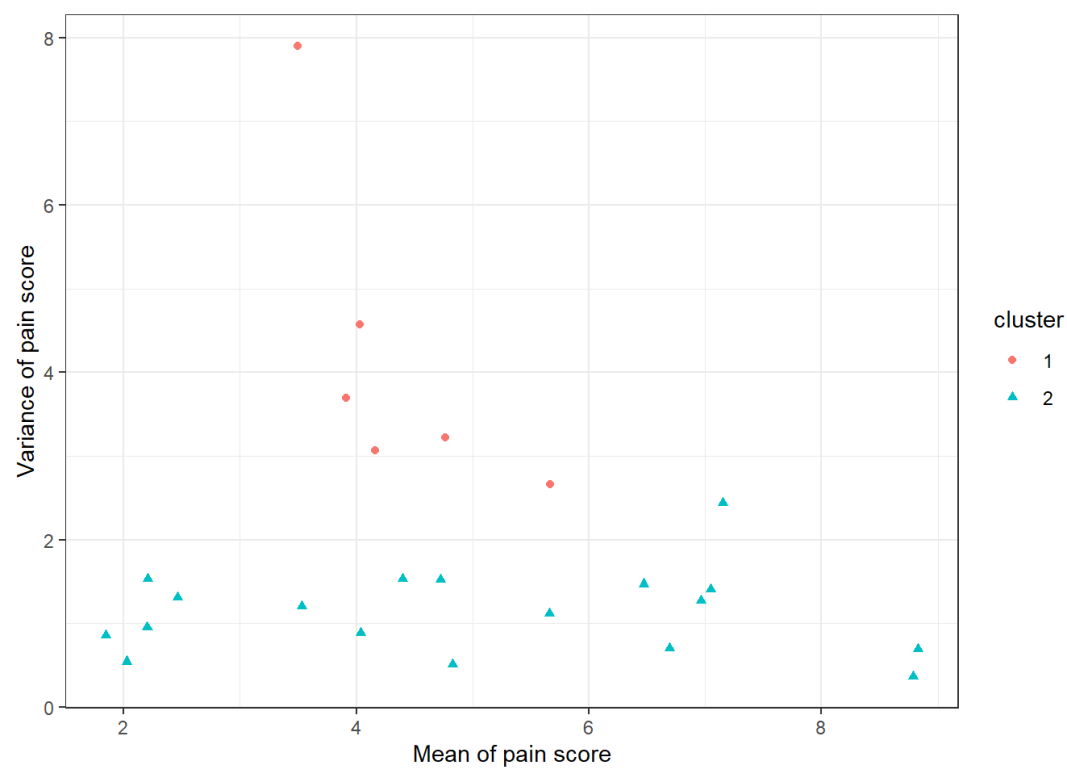

Figure A2: Two class model

Supplement: Supplementary data [file bmjopen-2022-062801supp002.pdf]
