# Supplementary material for: Day-to-day variability of knee pain and the relationship with physical activity in people with knee osteoarthritis: an observational, feasibility study using consumer smartwatches
Source: BMJ Open. 2023 Mar 13;13(3):e062801. doi: 10.1136/bmjopen-2022-062801 (PMC10016308; doi:10.1136/bmjopen-2022-062801)
Supplement: Author's manuscript [file bmjopen-2022-062801.draft_revisions.pdf]

BMJ Open

BMJ Open is committed to open peer review. As part of this commitment we make the peer review history of every article we publish publicly available.

When an article is published we post the peer reviewers' comments and the authors' responses online. We also post the versions of the paper that were used during peer review. These are the versions that the peer review comments apply to.

The versions of the paper that follow are the versions that were submitted during the peer review process. They are not the versions of record or the final published versions. They should not be cited or distributed as the published version of this manuscript.

BMJ Open is an open access journal and the full, final, typeset and author-corrected version of record of the manuscript is available on our site with no access controls, subscription charges or pay-per-view fees (http://bmjopen.bmj.com).

If you have any questions on BMJ Open's open peer review process please email info.bmjopen@bmj.com

BMJ Open

# Day to day variability of knee pain and the relationship with physical activity in people with knee osteoarthritis: A study using consumer smartwatches.

| | |
|---|---|
| Journal: | *BMJ Open* |
| Manuscript ID | bmjopen-2022-062801 |
| Article Type: | Original research |
| Date Submitted by the Author: | 22-Mar-2022 |
| Complete List of Authors: | Vivekanantham, Arani ; The University of Manchester; Oxford University Hospitals NHS Trust, Department of Rheumatology<br>Selby, David; The University of Manchester<br>Lunt, Mark; The University of Manchester<br>Sergeant, Jamie; The University of Manchester<br>Parkes, Matthew; The University of Manchester<br>O'Neill, Terence; The University of Manchester, Arthritis Research UK Centre for Epidemiology; Salford Royal NHS Foundation Trust, Department of Rheumatology<br>Dixon, Will; The University of Manchester; Manchester Academic Health Science Centre |
| Keywords: | PAIN MANAGEMENT, EPIDEMIOLOGY, Knee < ORTHOPAEDIC & TRAUMA SURGERY |
| | |

SCHOLARONE™
Manuscripts

*I, the Submitting Author has the right to grant and does grant on behalf of all authors of the Work (as defined in the below author licence), an exclusive licence and/or a non-exclusive licence for contributions from authors who are: i) UK Crown employees; ii) where BMJ has agreed a CC-BY licence shall apply, and/or iii) in accordance with the terms applicable for US Federal Government officers or employees acting as part of their official duties; on a worldwide, perpetual, irrevocable, royalty-free basis to BMJ Publishing Group Ltd ("BMJ") its licensees and where the relevant Journal is co-owned by BMJ to the co-owners of the Journal, to publish the Work in this journal and any other BMJ products and to exploit all rights, as set out in our licence.*

*The Submitting Author accepts and understands that any supply made under these terms is made by BMJ to the Submitting Author unless you are acting as an employee on behalf of your employer or a postgraduate student of an affiliated institution which is paying any applicable article publishing charge ("APC") for Open Access articles. Where the Submitting Author wishes to make the Work available on an Open Access basis (and intends to pay the relevant APC), the terms of reuse of such Open Access shall be governed by a Creative Commons licence – details of these licences and which Creative Commons licence will apply to this Work are set out in our licence referred to above.*

*Other than as permitted in any relevant BMJ Author's Self Archiving Policies, I confirm this Work has not been accepted for publication elsewhere, is not being considered for publication elsewhere and does not duplicate material already published. I confirm all authors consent to publication of this Work and authorise the granting of this licence.*

For peer review only

## Title page

# Day to day variability of knee pain and the relationship with physical activity in people with knee osteoarthritis: A study using consumer smartwatches.

Arani Vivekanantham [1, 2], David Selby [1], Mark Lunt [1], Jamie Sergeant [1], Matthew Parkes [1], Terence W O'Neill [1, 3, 4], William G Dixon [1, 3, 4]

[1] Centre for Epidemiology Versus Arthritis, University of Manchester, Manchester, UK.

[2] Department of Rheumatology, Nuffield Orthopaedic Centre, Oxford University Hospitals NHS Trust, Windmill Road, Oxford, UK.

[3] NIHR Manchester Biomedical Research Centre, Manchester University NHS Foundation Trust, Manchester Academic Health Science Centre, UK.

[4] Department of Rheumatology, Salford Royal NHS Foundation Trust, Salford, UK.

**Corresponding author**

Dr Arani Vivekanantham

Nuffield Department of Orthopaedics, Rheumatology and Musculoskeletal Sciences, Botnar Research Centre, Windmill Road, Oxford, OX3 7HD, UK.

arani.vivekanantham@ndorms.ox.ac.uk

**Email addresses for the co-authors:**

For peer review only

David Selby: david.selby@manchester.ac.uk, Mark Lunt: mark.lunt@manchester.ac.uk, Jamie Sergeant: jamie.sergeant@manchester.ac.uk, Matthew Parkes: matthew.parkes@manchester.ac.uk, Terence W O'Neill: terence.o'neill@manchester.ac.uk, William G Dixon: will.dixon@manchester.ac.uk

**Running title**

Pain and activity in knee osteoarthritis

For peer review only



For peer review only - http://bmjopen.bmj.com/site/about/guidelines.xhtml

## ABSTRACT

**Objective**: To conduct a feasibility study using smartwatches in people with knee osteoarthritis (OA) to determine the day-to-day variability of pain and the relationship between daily pain and step count.

**Design**: People aged >50 years with symptomatic knee OA were recruited and provided with a consumer cellular smartwatch with a bespoke app that triggered a series of daily questions including twice daily questions about level of knee pain and once monthly questions from the pain sub-scale of the Knee Injury and Osteoarthritis Outcome Score (KOOS) questionnaire. The smartwatch also recorded daily step counts.

**Results:** Of the 25 participants, 13 were male and their mean age was 65 years (standard deviation 8 years). The smartwatch app was successful in simultaneously assessing and recording data on knee pain and step count in real time. Knee pain could be categorized into sustained high/ low or fluctuating levels of pain, but there was considerable day to day variation within these categories. Levels of knee pain in general correlated with pain assessed by KOOS. Those with sustained high/ low levels of pain had a similar step count average, but those with fluctuating pain had much lower step count levels.

**Conclusions:** Smartwatches can be used to assess pain and physical activity in knee OA. Larger studies may help inform a better understanding of causal links between physical activity patterns and pain. In time, this could inform development of personalized physical activity recommendations for people with knee OA.


## Key words



For peer review only - http://bmjopen.bmj.com/site/about/guidelines.xhtml

Pain, knee osteoarthritis, mobile health (mHealth), patient-reported outcome, smartwatch.

**Strengths and limitations of this study**

- Our app was successful in assessing and recording data on patient reported symptoms and continuous sensor data simultaneously in real time.
- As this was a feasibility study, participant numbers were relatively small.
- Subjects volunteered for participation, and it is possible that selection factors and in particular an interest in smartwatches may have influenced engagement and reporting, limiting the generalization of the findings.
- Participants self-reported their knee OA; participants may have had varying severities of their knee OA (early disease to end-stage).
- The step count used was an estimate of the true step count levels, which was broken up by charging cycles- some activity may have been missed, leading to measurement error. Also, the total step count is a very rough measure of physical activity and misses patterns within the day.



For peer review only - http://bmjopen.bmj.com/site/about/guidelines.xhtml

## Introduction

Knee osteoarthritis (OA) affects one in eight people over the age of 60 years and pain is a cardinal symptom. Longitudinal studies suggest that the symptoms of knee OA typically follow relatively stable long-term trajectories [1, 2, 3], however, this has been based on infrequently collected data. It has more recently been proven that there is substantial day to day and within-day variability of pain in people with knee OA [1, 2, 3, 4]. Capturing such data presents challenges; clinical practice and most research studies capture pain reports at discrete time points, often months apart [5]. We thus have a limited picture of daily pain trajectories; such data are important because variations in pain, and its inherent uncertainty and unpredictability, can impact significantly on patients' quality of life.

Pain in knee OA may be influenced by a range of other factors including levels of physical activity [6, 7]. Like pain, physical activity assessment is also typically measured infrequently. Furthermore, it is often reliant on questionnaires which are subject to poor recall and poor precision. Understanding the relationship between pain and physical activity in knee OA has been limited by the ability to measure both variables simultaneously, accurately and in real time. The use of objectively measured levels of physical activity have been recommended [8, 9], yet only three known longitudinal studies have assessed the relationship between pain and steps per day in people with knee OA using such measures. One used an accelerometer to measure step count [10] and the other two used a research-grade activity monitor (StepWatch;



For peer review only - http://bmjopen.bmj.com/site/about/guidelines.xhtml

ActivPAL) [11, 12]. However, in all three studies pain levels were measured at different time points, in other words not simultaneously, to the physical activity.

Consumer technology including smartphones and wearables provides opportunity to measure both patient reported outcomes (such as pain) and passively collected sensor data (such as physical activity) contemporaneously. Smartphone apps are increasingly being used to track symptoms including in rheumatic diseases [13]. Although smartphones contain accelerometers allowing assessment of movement, they are not habitually carried by the user, and may thus misrepresent true patterns of activity [14]. Dedicated wearable devices like fitness trackers, often wrist-worn, can continuously track movement, but rarely support concurrent data entry.

In this observational feasibility study, called ‘Knee OsteoArthritis: Linking Activity and Pain’ (KOALAP), we developed a cellular smartwatch app to record and assess pain in real time whilst also collecting raw and processed sensor data allowing contemporaneous capture of physical activity.

## Aims

Using data from the KOALAP feasibility study, our aim was to:

1. To describe the day to day and within-day variability of pain intensity in people with symptomatic knee OA.



For peer review only - http://bmjopen.bmj.com/site/about/guidelines.xhtml

2. To assess how daily pain scores compare to pain assessed using monthly Knee Injury and Osteoarthritis Outcome Score (KOOS) pain domain scores.

3. To identify clusters of people with similar longitudinal pain scores.

4. To assess whether daily step count levels, assessed using the sensor data, are correlated with daily pain levels.

## METHODS

### Study design and sample

KOALAP was a longitudinal, observational feasibility smartwatch study in people with symptomatic knee OA. The full protocol for the KOALAP study has been published elsewhere [15]. In brief 26 participants with self-reported knee OA were recruited to the study in 2017. All participants were aged 50 years or over and either lived in or were willing to travel to the Greater Manchester area. The study was advertised in local newspapers, magazines and via social media channels. Interested participants were asked to contact the study team, after which they were sent a patient information sheet and an invitation to one of four enrollment events. The study involves human participants and was approved by the University of Manchester Research Ethics Committee (#0165).

### Data collection



For peer review only - http://bmjopen.bmj.com/site/about/guidelines.xhtml

122 At the enrollment event participants self-reported their age, gender, height, and weight. They were then provided with a consumer cellular smartwatch (Huawei Watch 2) with a bespoke app that collected i) patient-reported outcomes via questionnaires and ii) continuous watch sensor data. The participants were instructed to wear the watch from waking until going to bed and to answer the watch questions when prompted within a specific time window, illustrated in Table 1. All data were collected daily for 90 consecutive days.

Table 1: A summary of the frequency, trigger times and completion window times of the smartwatch questions.

| Question | Frequency | Trigger time | Window |
|---|---|---|---|
| Level of knee pain | Twice daily | 12.22 and 18.22 | 4 hours |
| 26 items from the Knee Injury and Osteoarthritis Outcome Score (KOOS) questionnaire | Monthly | Days 14, 44, 74 from start point | 1 week |

For the twice daily 'level of knee pain' questions participants were asked to record their level of knee pain on a 0-10 numeric rating scale [16]. To reduce responder burden, the KOALAP app used a subset of 26 items from the KOOS questionnaire in the app. This included the sub-scales on 'pain' and 'function, activities of daily living' (items P1-P9 and A1-A17 respectively illustrated in Appendix- TableA1). These sub-scales were felt to contain the items most directly relevant to



For peer review only - http://bmjopen.bmj.com/site/about/guidelines.xhtml

pain and function [17]. For this study, we decided to only include data from the ‘pain’ sub-scale (i.e., questions P2- P9 only). Participants were asked to score their responses on the following five-point scale: none (0), mild (1), moderate (2), severe (3) and extreme (4). We then took the sum of these item scores for each participant to give them a score for each month. We then scaled the score to a percentage, given the maximum possible score was 32, and divided by 10 to give us a score in the range between 1-10. We did not impute any missing data.

## Statistical methods

Descriptive statistics were used to describe participant characteristics including mean and standard deviation (SD) or median and interquartile range (IQR) for continuous data, and frequency (%) for categorical variables. Body mass index (BMI) was calculated from participants’ height (meters) divided by weight (kilograms) squared.

### *Questionnaire data*

We plotted twice daily pain scores over the 90-day period on individual graphs for each of the participants. On assessing data quality descriptively prior to the main analysis, we believed certain scores of 0 may have been submitted by accident. This was due to the user interface of the smartwatch app having a default value of zero and thus occasionally generating zero-values as an error in data input. Given this error in the data input, all pain scores of zero (of which there were 212 in total) were treated as missing values. Individual daily pain scores (numerical rating scale 1-10) were re-plotted, then visually reviewed for distribution patterns. The

For peer review only



For peer review only - http://bmjopen.bmj.com/site/about/guidelines.xhtml

difference between early evening and lunchtime pain scores for each day of the 90-day period was calculated to assess within-day variability in level of knee pain. Monthly KOOS pain scores for each participant were plotted on top of the day-to-day pain scores.

The mean and variance of the lunchtime and early evening pain scores were calculated for each participant over the whole study period, giving an indication of the average level of pain and its variability within each participant's time series. These participant-level summary statistics were passed into a k-means algorithm [18]. From the plotted graphs of trajectories of daily pain scores for each of the participants, three broad patterns of pain were identified: sustained high levels of pain, sustained low levels of pain and fluctuating levels of pain. Based on this clusters (k=3) were chosen for the algorithm; this grouped the participants into the three respective pain level groups. This was done separately for both the lunchtime and early evening pain; as the patterns of pain were similar, we decided to present only the lunchtime pain scores. In line with previous literature by Hawker and colleagues who have written extensively about the idea of OA causing two types of pain: intermittent (variable) and constant (stable) [19], we also explored a 2-class model (based on variance only) which included constant (stable) pain and intermittent (variable) pain.

*Sensor data*

Physical activity levels were assessed as total steps per day, measured by the app. The step counts were estimated from the accelerometry data using a proprietary algorithm. For our analysis, only estimated step counts over each calendar day were required. However, the data



For peer review only - http://bmjopen.bmj.com/site/about/guidelines.xhtml

from the smartwatch were not recorded as daily step counts; but rather a monotonically increasing number sequence that re-sets at irregular intervals, corresponding to times when the smartwatch was re-charged. To convert these data into daily step counts, we calculated the consecutive differences (i.e., the increases from one data point to the next recorded data point, for that person) of the raw counts (i.e., counts as recorded by the smartwatch) for each user. Where the *raw* differences were negative (i.e., when the raw count has re-set indicating the internal stepometer has re-set) or the time lag between consecutive data points were more than one day, the difference was re-set to zero. Resetting differences over more than one day to zero is important, because it is not known if the step count after such a gap is due to the accumulation of multiple days, or whether they were all from that single day. Therefore, it avoids spuriously high step counts for certain days. The sum of this modified sequence was then computed for each calendar date for each participant, giving the estimated daily step counts. We looked then at the mean and variance of step counts by pain cluster group. We looked also at the correlation between same day morning pain scores and step counts by participant using Pearson correlation.

All the statistical analyses were carried out using R version 3.6.0 [20]. The R packages dplyr [21] and tidyr [22] were used for data manipulation and the package ggplot2 [23] was used to produce the graphs.

**vi). Missing data**

For peer review only



For peer review only - http://bmjopen.bmj.com/site/about/guidelines.xhtml

We did not impute missing data.

## Patient and Public Involvement

Patients or the public were not involved in the design, or conduct, or reporting, or dissemination plans of our research.

## RESULTS

### Subject characteristics

A total of 26 participants were recruited. One participant left the study early and was excluded from the analysis. The mean age of the participants was 65 years, (SD= 8 years). Of the 25 participants, just over half (n=13) were male. The median (interquartile range) BMI was 27 (25, 35) kg/m$^2$.

### Daily knee pain

The twice daily questions were completed on around half of the participant-days [24]. In total, there were 2756 pain reports (out of a potential of 4680 pain reports), giving an average completion rate of approximately 59% for these questions. Of these, 78 were zero values which were then excluded from the analysis given the concern about false entries (see methods section and Figure A1 in the Appendix). Day-to-day patterns of knee pain, assessed at 12.22pm, for each of the 25 participants over the three-month study period is presented in Figure 1. The data show that there is a considerable amount of variation in the day-to-day levels of knee pain

For peer review only



For peer review only - http://bmjopen.bmj.com/site/about/guidelines.xhtml

in the participants. Some participants (for example participant IDs 3 and 12) had sustained high levels of pain, some participants (for example participant ID 18) had sustained low levels of pain, while others (for example, participant IDs 1, 2, 9, 11) had more fluctuating pain levels.

For the majority of participants (n=20) there was no overall difference between their daily 12.22pm and 18.22pm pain scores (median difference of zero (IQR 1)). In the remaining five participants, four reported worse pain in the early evening (participant IDs 9, 16, 24, 25) as illustrated graphically in Figure 2 below, which shows a Bland-Altman plot of self-reported daily scores for overall early evening versus overall lunchtime pain, by participant. For most participants, the mean difference is near zero. There are some users for whom early evening pain scores is slightly higher (for example, participant ID 9).

As there was no difference in daily pain scores between the two time points, in the future analysis included in this manuscript we focus only on the lunchtime pain scores.

**Monthly KOOS pain scores**

The monthly KOOS pain scores (where available) for each participant were plotted alongside their day-to-day levels of pain, as red dots, in Figure 1 above. It is not possible to directly compare the KOOS pain score and the daily level of pain, however, there were some interesting observations seen. For example, the KOOS pain scores were sometimes lower than that suggested by the daily pain scores (for example in participant IDs 3 and 10) and sometimes the



For peer review only - http://bmjopen.bmj.com/site/about/guidelines.xhtml

KOOS pain scores were higher than the daily pain scores (for example in participant IDs 4 and 18). For most of the participants the degree of variability in the KOOS pain scores within an individual was less marked than the day-to-day variability in pain though apart from perhaps one or two participants (for example, participant ID 3) tended to follow the broad pattern of daily pain over the 3 months.

**Cluster analysis**

The mean and variance of the lunchtime (as well as early evening) pain scores for each of the participants are shown in the appendix (in Table A2). Using the k algorithm and including k-3 subjects were characterized into clusters (see Figure 3). On review of the lunchtime pain patterns (as shown in Figure 1 above) suggested three broad clusters of pain: high, low, and fluctuating levels of pain.

As mentioned in the methods section, we also explored a 2-class model (based on variance only) which included constant (stable) pain and intermittent (variable) pain. Our findings of this are illustrated in Figure A2 in the Appendix. We decided to focus on the 3-cluster model in this paper and present the above methods in the main paper.

**Physical activity/ step count data**



For peer review only - http://bmjopen.bmj.com/site/about/guidelines.xhtml

262 The estimated daily step count levels for each of the 25 participants over the three-month period is shown in Figure 4. The figure highlights the variability in step counts with some participants having little variability (for example, participant IDs 11 and 12), others having high variability (for example, participant IDs 24 and 25) and one (participant ID 4) having two very distinct patterns of activity.

268 To explore whether the differences in step count levels between the participants was linked to their daily pain levels, we calculated the mean (and standard deviation) step count per day for the three clusters, as shown in Table 2. We found that those with sustained high or sustained low pain levels had a similar step count level, however, those with fluctuating pain levels (i.e., unpredictable levels of pain) had much lower step count levels.

Table 2: Mean and standard deviation of daily step count by pain cluster.

| **Pain cluster** | | |
|---|---|---|
| | **Mean** | **Standard deviation** |
| Sustained high pain levels | 3754 | 2524 |
| Sustained low pain levels | 4307 | 2992 |
| Fluctuating pain levels | 2064 | 1716 |



For peer review only - http://bmjopen.bmj.com/site/about/guidelines.xhtml

In an individual analysis, for the majority of participants (n=17) the correlation coefficients were positive indicating that increased step count was linked with increasing pain however the correlation coefficients were small, confidence intervals were wide and embraced unity, as illustrated in Table A3 in the appendix. In some participants who had sustained high lunchtime pain scores, step count levels were low (for example, participant ID 3). In other participants who had fluctuating pain scores (ranging from low to high), some had fluctuating step count levels (ranging from 0 steps to > 20,000) (for example, participant IDs 1, 24, 25) whereas others had sustained low step counts (for example, participant IDs 2, 9 ,11) irrespective of high or low pain scores, as shown in Figure 5.

## DISCUSSION

We have shown that the use of our bespoke app on a consumer cellular smartwatch was successful in assessing and recording data on patient reported symptoms and continuous sensor data simultaneously in real time.

In our study, among people with knee OA we found that there was little within day but significant between day variability in pain. We found that when we categorised pain into three groups, patients separated into intermittent (variable) and constant, either sustained high or low levels of pain, supporting what is already known [25, 26, 27]. We also found that there was no difference in pain scores between the lunchtime and early evening scores in most of our participants though it may be that the app timings (notifications at 12.22 and 18.22) were not



For peer review only - http://bmjopen.bmj.com/site/about/guidelines.xhtml

truly representative of the maximal possible range of pain from morning to evening. Our study also found that participants with seemingly stable monthly KOOS pain scores had a lot of day-to-day variability in pain levels in between their monthly scores.

This unpredictable nature of pain in knee OA can be distressing for people [27] and can often lead to increased healthcare care use and reduced work productivity. In a study by Thomas et al (2021), it was reported that acute flares in people with knee OA can go on to last 3-8 days, with two-thirds of people experiencing increased stiffness alongside their pain. In this study acute flares were triggered by multiple activity-related, psychosocial, and environmental factors [28].

Moreover, interestingly, our study also found that those with either sustained high or low levels of pain have a similar step count average but those with fluctuating pain have much lower levels of activity. This may indicate that fluctuating pain (i.e., pain that is unpredictable/ always changing) may be more troublesome to people living with knee OA than those with, for example, sustained high levels of pain. Our finding that low levels of pain was not necessarily associated with higher step count levels highlights that there may be other factors related to low physical activity levels in people with knee OA (not just pain). It is also important to note the possible bidirectional nature of pain and physical activity, whereby low pain may enable greater activity, but also where increased activity may precipitate higher pain. Our analysis of this feasibility study did not examine this in detail, yet it provides evidence this might be possible in the future given the ability to collect temporally rich data.



For peer review only - http://bmjopen.bmj.com/site/about/guidelines.xhtml

Studies to date remain ambiguous regarding the link between the pain in knee OA and physical activity, with some studies finding a positive association [29] and some studies no association [10]. These differences in findings may in part be due to the different methods used to measure physical activity and the timings when the level of pain was assessed. For example, in a study by Parry et al, paper diaries were used to collect information on daily pain in people with knee OA, to assess the impact of specific physical exposures on increases in daily pain levels [29]. However, as these paper diaries were completed retrospectively, they may have been subject to errors of inaccurate recall. With the use of this novel method of using consumer devices to measure symptoms and step count concurrently, it may help provide a more accurate assessment of these associations in the future.

There are, however, also several limitations to be considered when interpreting the findings from this study. This was a feasibility study and so the participant numbers were relatively small. Subjects volunteered for participation, and it is possible that selection factors and in particular an interest in smartwatches may have influenced engagement and reporting, limiting the generalization of the findings. Participants self-reported their knee OA; this diagnosis was not confirmed with their medical records and participants may also have had varying severities of their knee OA (early disease to end-stage). Moreover, due to the user interface of the smartwatch app having a default value of zero and thus occasionally generating zero-values as

For peer review only



For peer review only - http://bmjopen.bmj.com/site/about/guidelines.xhtml

an error in data input, all zero values were treated as missing values to avoid 'false' zeros; we recognize that in doing this we may have also removed some 'true' zeros.

There are also several limitations related to the step-count. The watch measured accelerometry data and a proprietary algorithm estimated the number of steps from these data. However, in a study that compared commercially available activity tracking devices for step count accuracy no significant differences were found among the devices [30]. The step count used was an estimate of the true step count levels, which was broken up by charging cycles- some activity may have been missed, leading to measurement error. Also, the total step count is a very rough measure of physical activity and misses patterns within the day [30]. Moreover, the qualitative details, such as whether the walking was slow/ fast (which have stronger associations with pain), was missed. We do, however, have the raw accelerometer data and will conduct future analysis to help us to better understand the relationship between pain and more granular patterns of physical activity.

It is well known that physical activity and exercise play a critical role in the conservative management of knee OA [31], however physical activity based exposures may also trigger acute flares. Therefore, a personalized approached to physical activity in knee OA is recommended, adjusted to the patient's phenotype [32]. In the future, the use of consumer devices may aid this personalized approach as it will help to identify the optimal level of physical activity for



For peer review only - http://bmjopen.bmj.com/site/about/guidelines.xhtml

each patient before it triggers an exacerbation of pain and could potentially provide personalised (digital) coaching.

In summary, this feasibility study has shown that the use of the consumer cellular smartwatch app was successful in assessing and recording data on patient reported outcomes and continuous sensor data simultaneously in real time. This data allowed us to highlight the substantial variability in knee pain due to OA and its links with physical activity. Larger studies are required to confirm our findings and allow exploration of the complex bidirectional relationships between activity and pain. This method of data capture should help to develop personalized physical activity recommendations for people with knee OA in the future, based on what their optimal level of physical activity is in relation to their pain.



For peer review only - http://bmjopen.bmj.com/site/about/guidelines.xhtml

## Author contributions

WGD, TON, JS, and MP were involved in the conception, design, and data collection for the study. DS and AV were involved in the data analysis and all authors were involved in the interpretation of the results. AV drafted the article with critical revision from all authors. All authors approved the final version of the article to be published.

## Acknowledgements

This project has been possible through collaboration with the Google Fit Research team at Google UK. The Google team has collaboratively built the KOALAP app for self-reported data collection and the system for collecting and transmitting sensor data.

## Funding

The study was funded by Arthritis Research UK (grants 21225 and 21755) and supported by the NIHR Manchester Biomedical Research Centre (grant number N/A). AV is supported by a National Institute for Health and Research (NIHR) funded Academic Clinical Fellowship (grant number N/A). The views expressed in this publication are those of the author(s) and not necessarily those of the NHS, the NIHR or the Department of Health.

## Conflicts of interest

WGD has received consultancy fees from Google and Abbvie.

## Data Sharing Statement

All data relevant to the study are included in the article and uploaded as supplementary information**.**

For peer review only



For peer review only - http://bmjopen.bmj.com/site/about/guidelines.xhtml

## Ethics approval

The study involves human participants and was approved by the University of Manchester Research Ethics Committee (#0165).

## References


1. Soni A, Kiran A, Hart DJ, et al. Prevalence of reported knee pain over twelve years in a community-based cohort. *Arthritis & Rheumatism*. 2012;64(4):1145-1152. doi:10.1002/art.33434
2. Cedraschi C, Delézay S, Marty M, et al. “Let’s Talk about OA Pain”: A Qualitative Analysis of the Perceptions of People Suffering from OA. Towards the Development of a Specific Pain OA-Related Questionnaire, the Osteoarthritis Symptom Inventory Scale (OASIS). Milanese S, ed. *PLoS ONE*. 2013;8(11):e79988. doi:10.1371/journal.pone.0079988
3. Schneider S, Junghaenel DU, Keefe FJ, Schwartz JE, Stone AA, Broderick JE. Individual differences in the day-to-day variability of pain, fatigue, and well-being in patients with rheumatic disease: Associations with psychological variables. *Pain*. 2012;153(4):813-822. doi:10.1016/j.pain.2012.01.001
4. Parry E, Ogollah R, Peat G. Significant pain variability in persons with, or at high risk of, knee osteoarthritis: preliminary investigation based on secondary analysis of cohort data. *BMC Musculoskelet Disord*. 2017;18(1):80. doi:10.1186/s12891-017-1434-3
5. Allen KD, Coffman CJ, Golightly YM, Stechuchak KM, Keefe FJ. Daily pain variations among patients with hand, hip, and knee osteoarthritis. *Osteoarthritis Cartilage*. 2009;17(10):1275-1282. doi:10.1016/j.joca.2009.03.021


For peer review only



For peer review only - http://bmjopen.bmj.com/site/about/guidelines.xhtml

6. Parry E, Ogollah R, Peat G. "Acute flare-ups" in patients with, or at high risk of, knee osteoarthritis: a daily diary study with case-crossover analysis. *Osteoarthritis Cartilage*. 2019;27(8):1124-1128. doi:10.1016/j.joca.2019.04.003

7. Thomas MJ, Butler-Walley S, Rathod-Mistry T, et al. Acute flares of knee osteoarthritis in primary care: a feasibility and pilot case-crossover study. *Pilot Feasibility Stud*. 2018;4(1):167. doi:10.1186/s40814-018-0359-4

8. Prince SA, Adamo KB, Hamel M, Hardt J, Connor Gorber S, Tremblay M. A comparison of direct versus self-report measures for assessing physical activity in adults: a systematic review. *Int J Behav Nutr Phys Act*. 2008;5(1):56. doi:10.1186/1479-5868-5-56

9. Verlaan L, Bolink SAAN, Van Laarhoven SN, et al. Accelerometer-based Physical Activity Monitoring in Patients with Knee Osteoarthritis: Objective and Ambulatory Assessment of Actual Physical Activity During Daily Life Circumstances. *TOBEJ*. 2015;9(1):157-163. doi:10.2174/1874120701509010157

10. Brisson NM, Gatti AA, Maly MR. Association of Pain and Steps Per Day in Persons With Mild-to-Moderate, Symptomatic Knee Osteoarthritis: A Mixed-Effects Models Analysis of Multiple Measurements Over Three Years. *Arthritis Care Res*. 2020;72(1):114-121. doi:10.1002/acr.23842

11. White DK, Tudor-Locke C, Zhang Y, et al. Prospective change in daily walking over 2 years in older adults with or at risk of knee osteoarthritis: the MOST study. *Osteoarthritis and Cartilage*. 2016;24(2):246-253. doi:10.1016/j.joca.2015.08.004



For peer review only - http://bmjopen.bmj.com/site/about/guidelines.xhtml

12. Parkes MJ, Jones RK, Carter SC, Liu A, Callaghan MJ, Felson DT. Change in pain and its relation to change in activity in osteoarthritis. *Osteoarthritis and Cartilage Open*. 2020;2(3):100063. doi:10.1016/j.ocarto.2020.100063

13. Dixon WG, Michaud K. Using technology to support clinical care and research in rheumatoid arthritis. *Current Opinion in Rheumatology*. 2018;30(3):276-281. doi:10.1097/BOR.0000000000000485

14. Silva AG, Simões P, Queirós A, Rodrigues M, Rocha NP. Mobile Apps to Quantify Aspects of Physical Activity: a Systematic Review on its Reliability and Validity. *J Med Syst*. 2020;44(2):51. doi:10.1007/s10916-019-1506-z

15. Beukenhorst AL, Parkes MJ, Cook L, et al. Collecting Symptoms and Sensor Data With Consumer Smartwatches (the Knee OsteoArthritis, Linking Activity and Pain Study): Protocol for a Longitudinal, Observational Feasibility Study. *JMIR Res Protoc*. 2019;8(1):e10238. doi:10.2196/10238

16. Turk DC, Dworkin RH, Burke LB, et al. Developing patient-reported outcome measures for pain clinical trials: IMMPACT recommendations. *Pain*. 2006;125(3):208-215. doi:10.1016/j.pain.2006.09.028

17. Roos EM, Roos HP, Lohmander LS, Ekdahl C, Beynnon BD. Knee Injury and Osteoarthritis Outcome Score (KOOS)—Development of a Self-Administered Outcome Measure. *J Orthop Sports Phys Ther*. 1998;28(2):88-96. doi:10.2519/jospt.1998.28.2.88

18. Hartigan JA, Wong MA. Algorithm AS 136: A K-Means Clustering Algorithm. *Applied Statistics*. 1979;28(1):100. doi:10.2307/2346830

For peer review only



For peer review only - http://bmjopen.bmj.com/site/about/guidelines.xhtml

452 19. Carlesso LC, Hawker GA, Torner J, et al. Association of Intermittent and Constant Knee Pain Patterns With Knee Pain Severity and With Radiographic Knee Osteoarthritis Duration and Severity. *Arthritis Care Res (Hoboken)*. 2021;73(6):788-793. doi:10.1002/acr.24194

456 20. R Core Team. *R: A Language and Environment for Statistical Computing. R Foundation for Statistical Computing, Vienna, Austria.*; 2019. https://www.R-project.org/.

458 21. Wickham H, François R, Henry L, Müller K. *Dplyr: A Grammar of Data Manipulation.*; 2020. https://CRAN.R-project.org/package=dplyr

460 22. Wickham H, Henry L. *Tidyr: Tidy Messy Data.*; 2020. https://CRAN.R-project.org/package=tidyr

462 23. Wickham H. *Ggplot2: Elegant Graphics for Data Analysis.*; 2016.

463 24. Beukenhorst AL, Howells K, Cook L, et al. Engagement and Participant Experiences With Consumer Smartwatches for Health Research: Longitudinal, Observational Feasibility Study. *JMIR Mhealth Uhealth*. 2020;8(1):e14368. doi:10.2196/14368

466 25. Thomas MJ, Rathod-Mistry T, Harper S, et al. Acute Flares of Knee Osteoarthritis (the ACT-FLARE Study): Protocol for a Web-Based Case-Crossover Study in Community-Dwelling Adults. *JMIR Res Protoc*. 2019;8(4):e13428. doi:10.2196/13428

469 26. Thomas MJ, Butler-Walley S, Rathod-Mistry T, et al. Acute flares of knee osteoarthritis in primary care: a feasibility and pilot case-crossover study. *Pilot Feasibility Stud*. 2018;4(1):167. doi:10.1186/s40814-018-0359-4



For peer review only - http://bmjopen.bmj.com/site/about/guidelines.xhtml

27. Hawker GA, Stewart L, French MR, et al. Understanding the pain experience in hip and knee osteoarthritis – an OARSI/OMERACT initiative. *Osteoarthritis and Cartilage*. 2008;16(4):415-422. doi:10.1016/j.joca.2007.12.017

28. Thomas MJ, Rathod-Mistry T, Parry EL, Pope C, Neogi T, Peat G. Triggers for acute flare in adults with, or at risk of, knee osteoarthritis: a web-based case-crossover study in community-dwelling adults. *Osteoarthritis and Cartilage*. 2021;29(7):956-964. doi:10.1016/j.joca.2021.04.007

29. Parry E, Ogollah R, Peat G. 'Acute flare-ups' in patients with, or at high risk of, knee osteoarthritis: a daily diary study with case-crossover analysis. *Osteoarthritis and Cartilage*. 2019;27(8):1124-1128. doi:10.1016/j.joca.2019.04.003

30. Gaz DV, Rieck TM, Peterson NW, et al. Determining the Validity and Accuracy of Multiple Activity-Tracking Devices in Controlled and Free-Walking Conditions. *Am J Health Promot*. 2018;32(8):1671-1678. doi:10.1177/0890117118763273

31. Uthman OA, van der Windt DA, Jordan JL, et al. Exercise for lower limb osteoarthritis: systematic review incorporating trial sequential analysis and network meta-analysis. *BMJ*. 2013;347(sep20 1):f5555-f5555. doi:10.1136/bmj.f5555

32. Gay C, Chabaud A, Guilley E, Coudeyre E. Educating patients about the benefits of physical activity and exercise for their hip and knee osteoarthritis. Systematic literature review. *Ann Phys Rehabil Med*. 2016;59(3):174-183. doi:10.1016/j.rehab.2016.02.005

For peer review only



For peer review only - http://bmjopen.bmj.com/site/about/guidelines.xhtml

For peer review only



For peer review only - http://bmjopen.bmj.com/site/about/guidelines.xhtml

**Appendix**

The tables and figures in the appendix have been submitted as separate documents, as per the journal’s requirements.

For peer review only



For peer review only - http://bmjopen.bmj.com/site/about/guidelines.xhtml

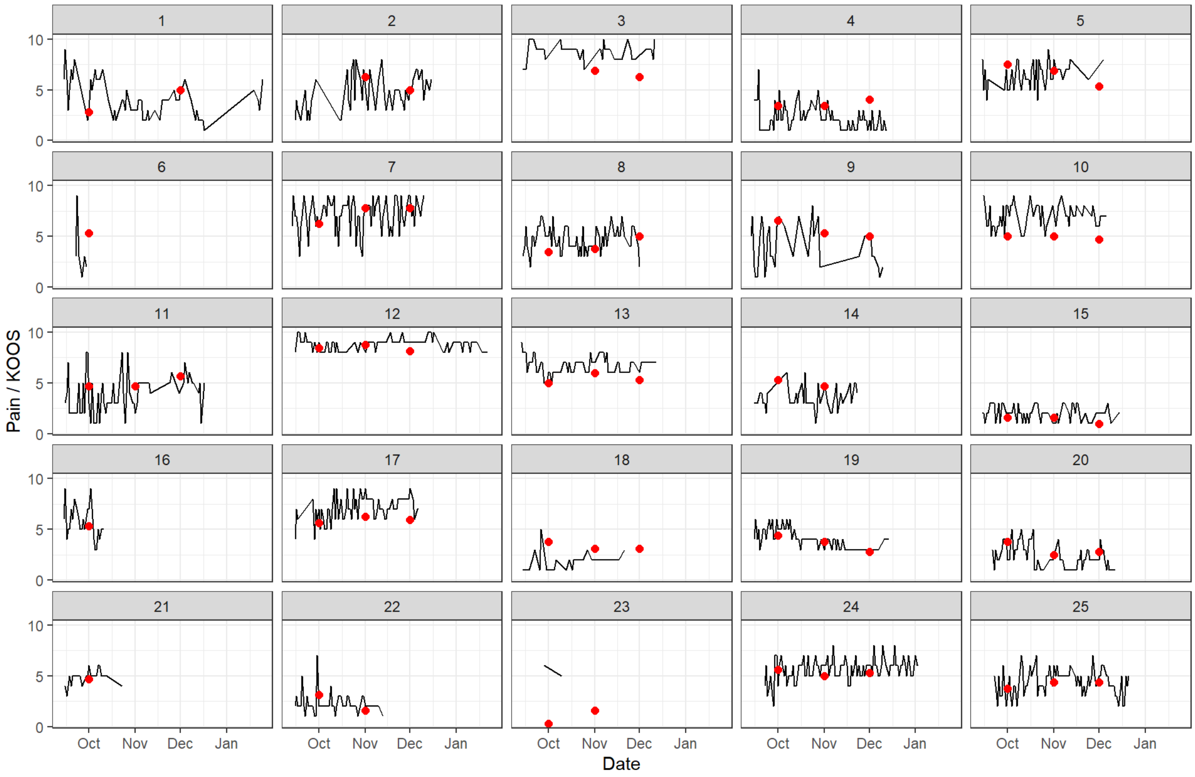


Figure 1: Daily pain scores (assessed at 12.22pm) and monthly Knee Injury and Osteoarthritis Outcome Score (KOOS) scores, represented by the red dots, by participant ID.

For peer review only

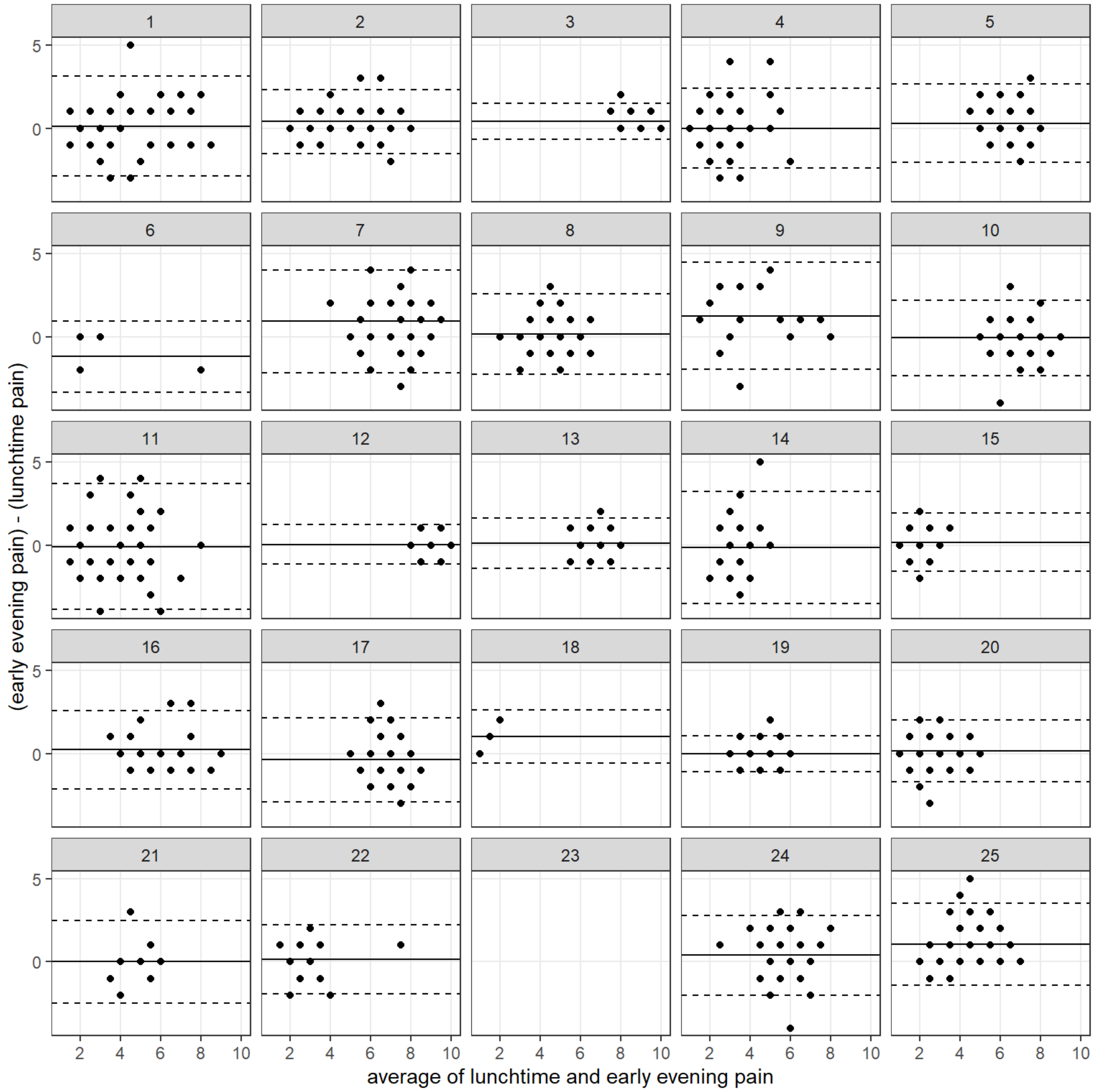


Figure 2: Bland-Altman plot of self-reported daily scores for overall early evening versus overall lunchtime pain, by participant. The black line is the mean difference, the dashed lines are +/- 1.96 * SD (difference).

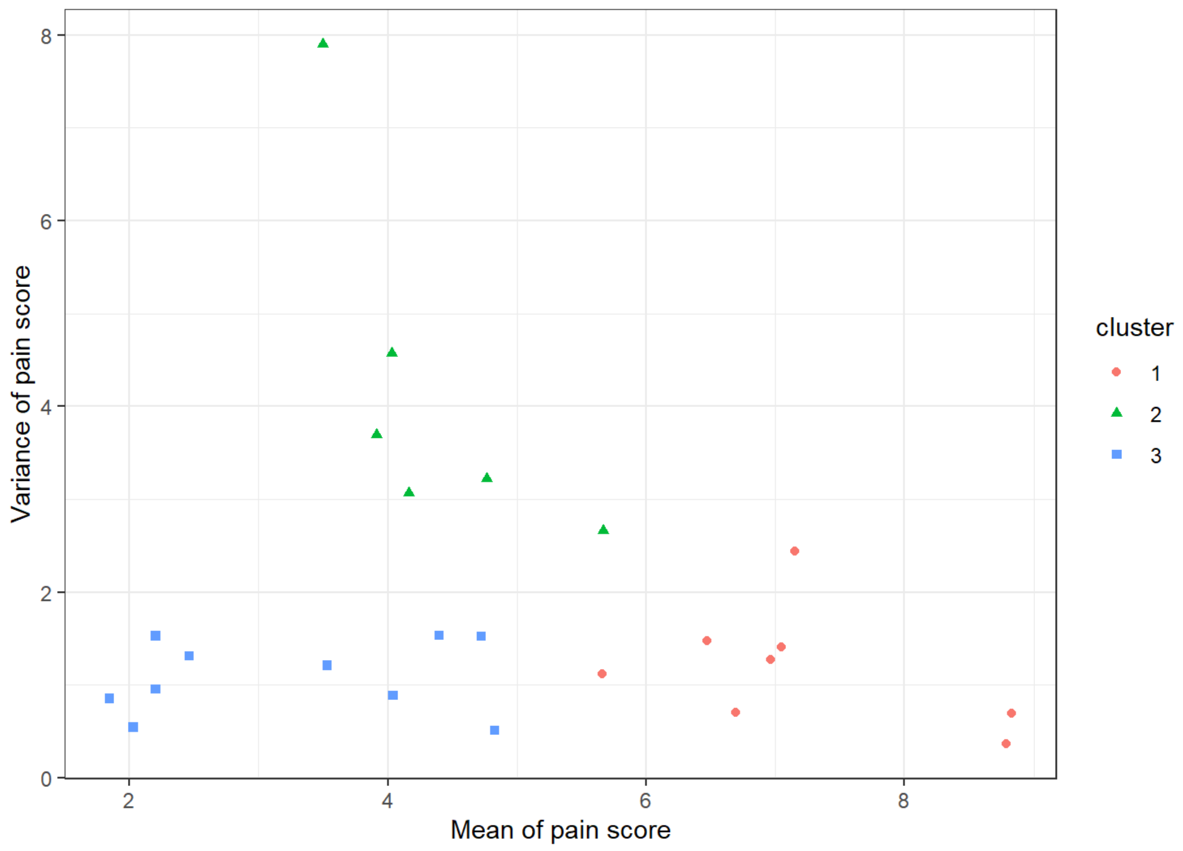


Figure 3: Variance of lunchtime pain by mean pain score with each point representing a pain cluster. The red circles (cluster 1) represent users with sustained high levels of pain, the blue squares (cluster 3) represent those with sustained low levels of pain, and the green triangles (cluster 2) represent those with fluctuating pain levels.

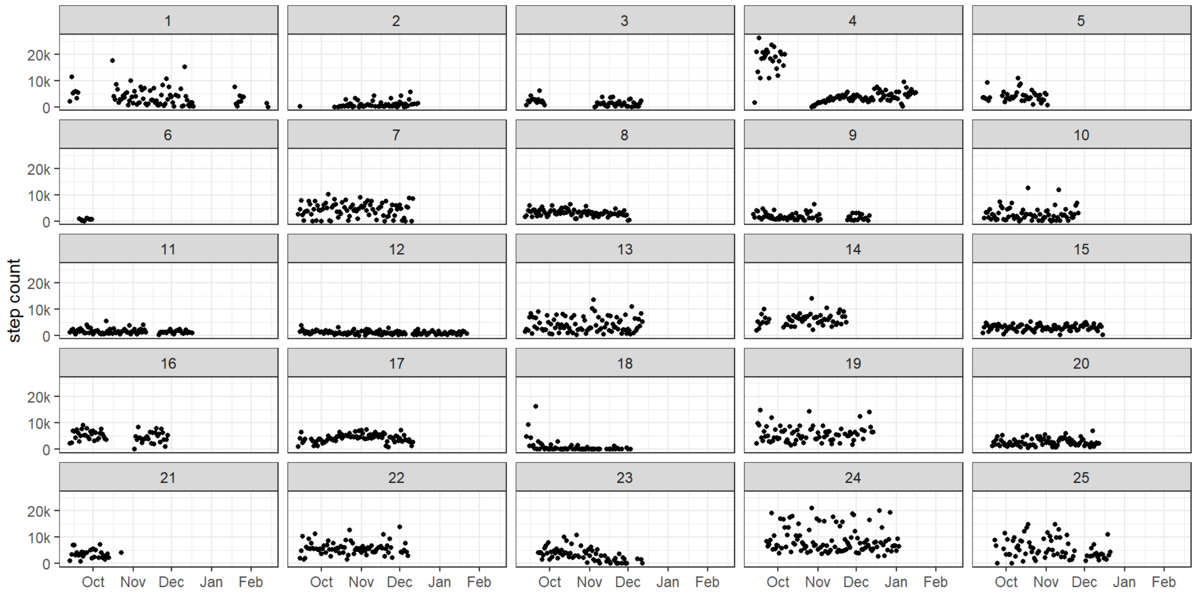


Figure 4: Daily step count levels by participant ID over the three-month study period.

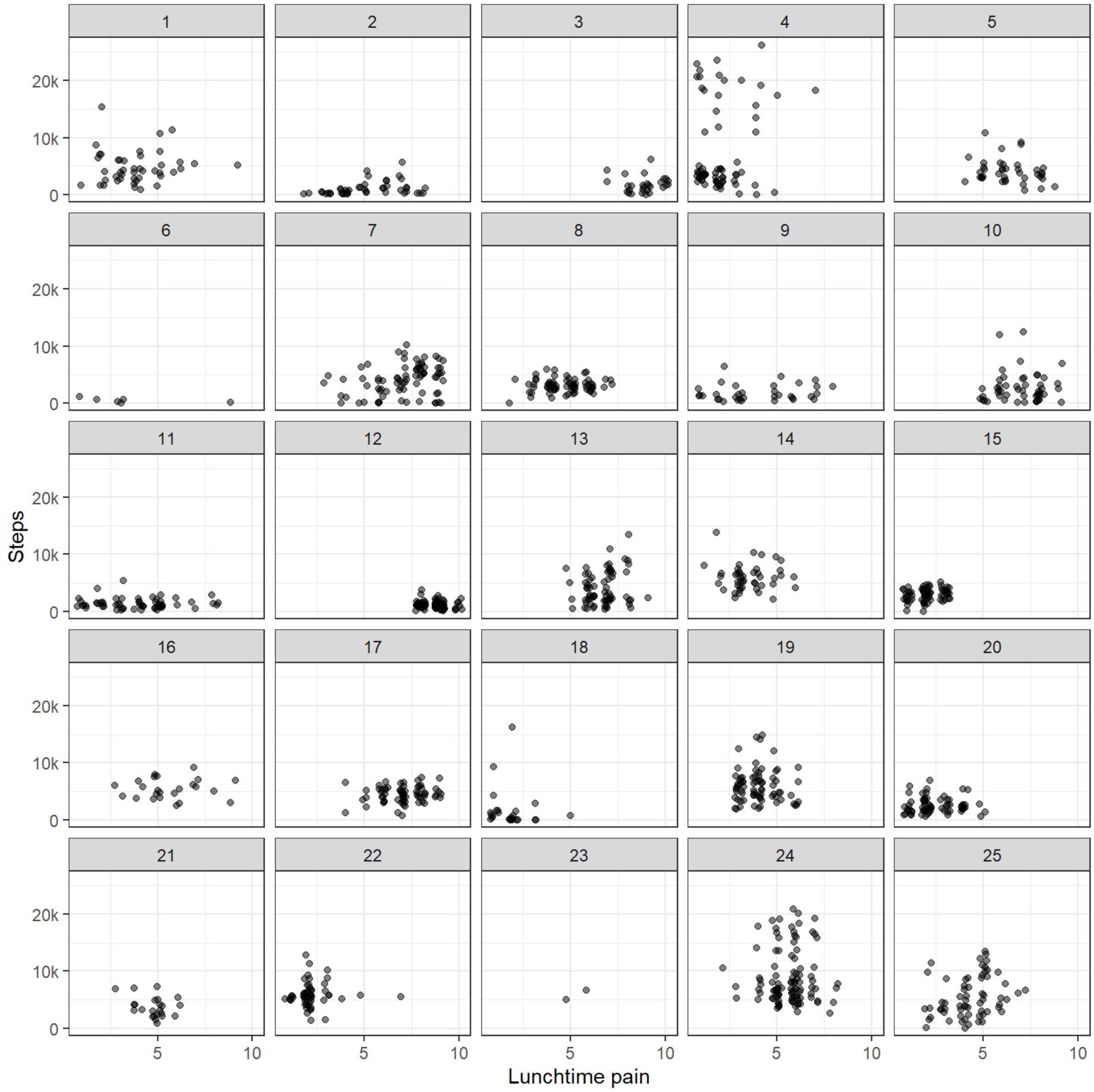


Figure 5: Jittered scatter plot of daily step count level against overall lunchtime pain, by participant.

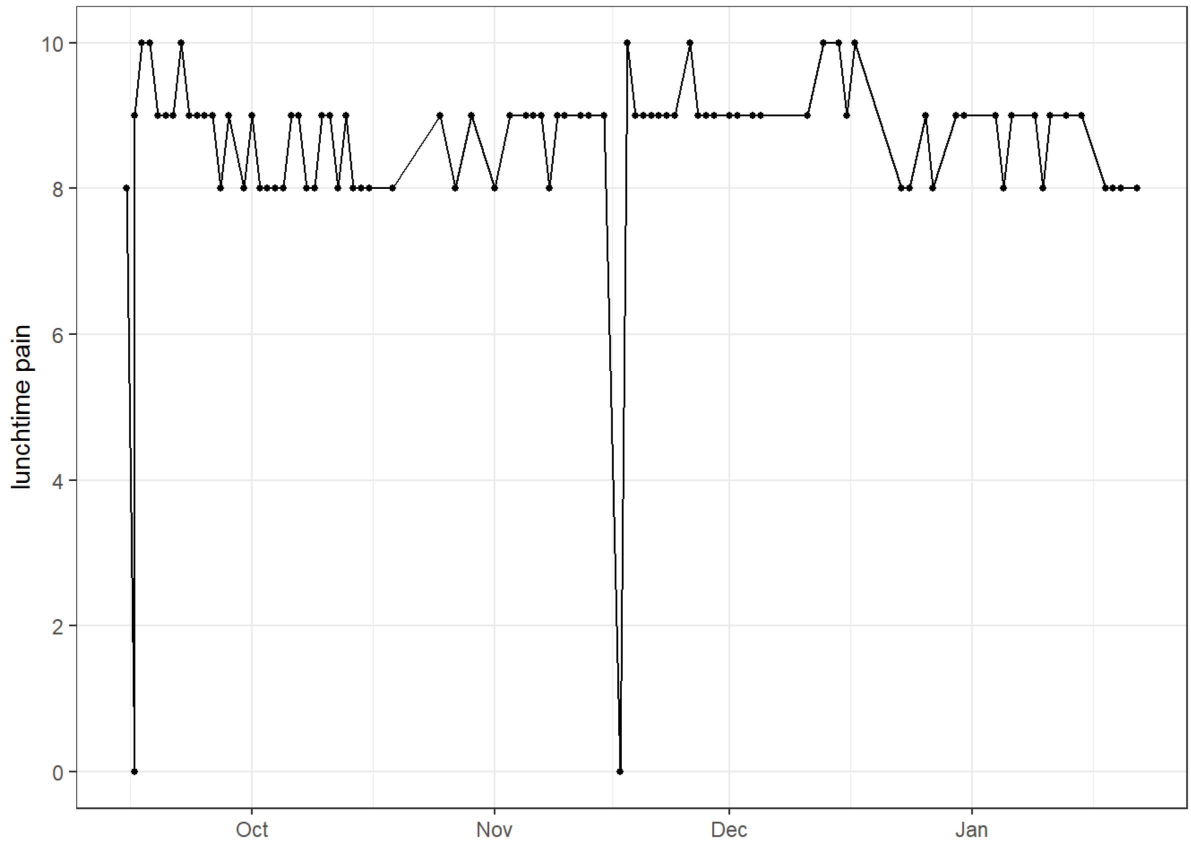


Figure A1: An example of a user with what we believe shows ‘false zeros’ (i.e., entered by mistake due to user interface error)

view only

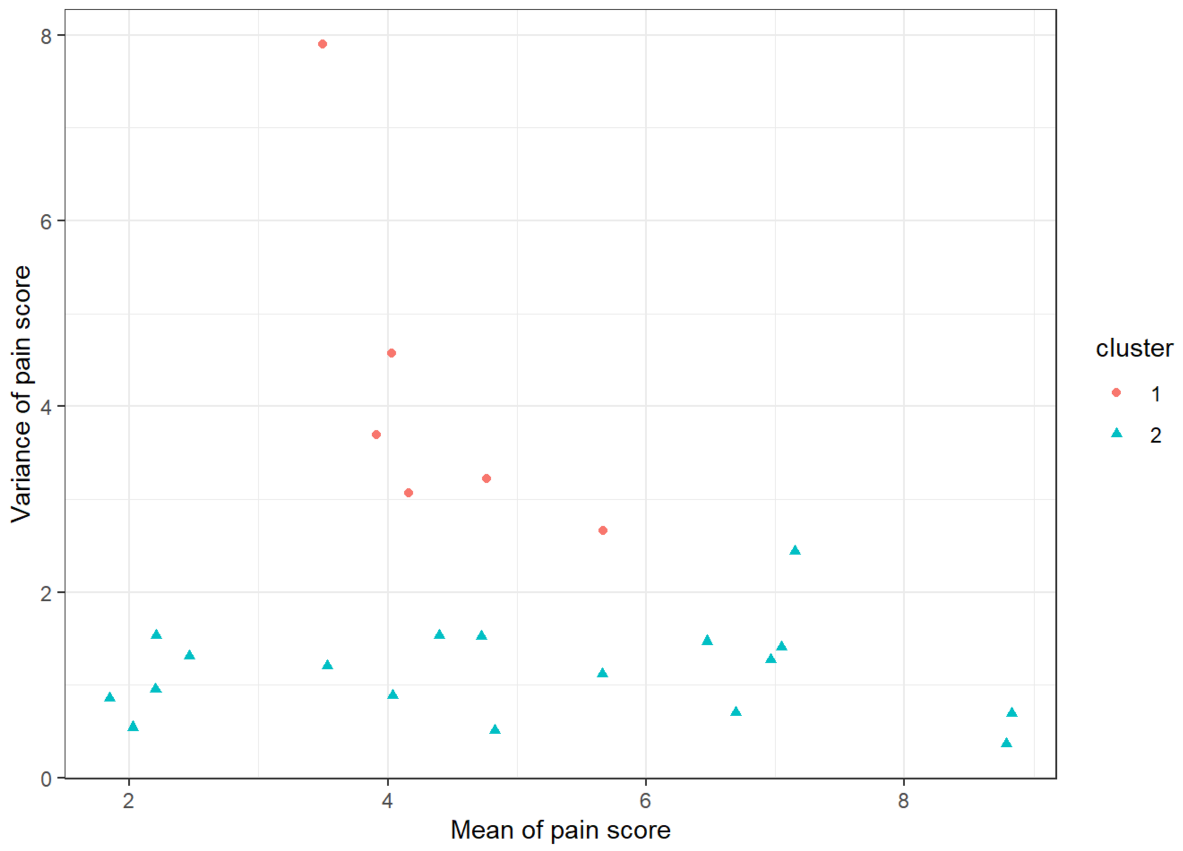


Figure A2: Two class model

review only

Table A1: 17 questions from the Knee Injury and Osteoarthritis Outcome Score (KOOS) questionnaire that was used in this study.

| Sub-scale | Component | Response |
|---|---|---|
| **PAIN** | | |
| P1 | How often do you experience knee pain? | None/ Mild/ Moderate/ Severe/ Extreme |
| *What amount of knee pain have you experienced the last week during the following activities?* | | |
| P2 | Twisting/pivoting on your knee | None/ Mild/ Moderate/ Severe/ Extreme |
| P3 | Straightening knee fully | |
| P4 | Bending knee fully | |
| P5 | Walking on flat surface | |
| P6 | Going up or down stairs | |
| P7 | At night while in bed | |
| P8 | Sitting or lying | |
| P9 | Standing upright | |
| **FUNCTION, DAILY LIVING** *The following questions concern your physical function. By this we mean your ability to move around and to look after yourself. For each of the following activities please indicate the degree of difficulty you have experienced in the last week due to your knee.* | | |
| A1 | Descending stairs | None/ Mild/ Moderate/ Severe/ Extreme |
| A2 | Ascending stairs | |

For peer review only



For peer review only - http://bmjopen.bmj.com/site/about/guidelines.xhtml

| | | |
|---|---|---|
| *For each of the following activities please indicate the degree of difficulty you have experienced in the last week due to your knee.* | | |
| A3 | Rising from sitting | None/ Mild/ Moderate/ Severe/ Extreme |
| A4 | Standing | |
| A5 | Bending to floor/pick up an object | |
| A6 | Walking on flat surface | |
| A7 | Getting in/out of car | |
| A8 | Going shopping | |
| A9 | Putting on socks/stockings | |
| A10 | Rising from bed | |
| A11 | Taking off socks/stockings | |
| A12 | Lying in bed (turning over, maintaining knee position) | |
| A13 | Getting in/out of bath | |
| A14 | Sitting | |
| A15 | Getting on/off toilet | |
| *For each of the following activities please indicate the degree of difficulty you have experienced in the last week due to your knee* | | |
| A16 | Heavy domestic duties (moving heavy boxes, scrubbing floors, etc) | None/ Mild/ Moderate/ Severe/ Extreme |
| A17 | Light domestic duties (cooking, dusting, etc) | |

3

For peer review only



For peer review only - http://bmjopen.bmj.com/site/about/guidelines.xhtml

Table A2: Mean and variances of pain score by Participant ID

| Participant ID | Mean lunchtime pain level | Standard deviation lunchtime pain level | Mean early evening pain level | Standard deviation early evening pain level |
|---|---|---|---|---|
| 1 | 4.2 | 1.8 | 4.3 | 2.1 |
| 2 | 4.8 | 1.8 | 5.2 | 1.9 |
| 3 | 8.8 | 0.8 | 9.2 | 0.6 |
| 4 | 2.2 | 1.2 | 2.3 | 1.4 |
| 5 | 6.5 | 1.2 | 6.7 | 0.9 |
| 6 | 3.5 | 2.8 | 2.8 | 2.5 |
| 7 | 7.2 | 1.6 | 8.1 | 1.4 |
| 8 | 4.7 | 1.2 | 4.5 | 1.3 |
| 9 | 4.0 | 2.1 | 5.0 | 2.0 |
| 10 | 7.0 | 1.1 | 6.8 | 1.1 |
| 11 | 3.9 | 1.9 | 4.2 | 2.1 |
| 12 | 8.8 | 0.6 | 8.9 | 0.5 |
| 13 | 6.7 | 0.8 | 6.7 | 0.8 |

For peer review only

| | | | | |
|---|---|---|---|---|
| 14 | 3.5 | 1.1 | 3.4 | 1.2 |
| 15 | 2.0 | 0.7 | 2.2 | 0.9 |
| 16 | 8.1 | 0.6 | 8.2 | 0.7 |
| 17 | 5.7 | 1.6 | 5.9 | 1.7 |
| 18 | 7.0 | 1.2 | 6.9 | 1.1 |
| 19 | 1.8 | 0.9 | 2.6 | 1.2 |
| 20 | 4.0 | 0.9 | 4.1 | 0.9 |
| 21 | 2.5 | 1.1 | 2.6 | 1.1 |
| 22 | 4.8 | 0.7 | 4.9 | 1.0 |
| 23 | 2.2 | 1.0 | 2.8 | 1.4 |
| 24 | 5.7 | 1.1 | 6.0 | 1.0 |
| 25 | 4.4 | 1.2 | 5.5 | 1.3 |

For peer review only

Table A3: Pearson correlation between same-day lunchtime pain scores and step count by participant ID

| Participant ID | Pearson correlation |
|---|---|
| 1 | 0.05 |
| 2 | 0.35 |
| 3 | 0.00 |
| 4 | 0.17 |
| 5 | -0.21 |
| 6 | -0.62 |
| 7 | 0.27 |
| 8 | 0.09 |
| 9 | 0.11 |
| 10 | 0.02 |
| 11 | -0.01 |
| 12 | -0.12 |
| 13 | 0.18 |
| 14 | -0.05 |
| 15 | 0.23 |
| 16 | 0.06 |
| 17 | 0.16 |

For peer review only

| | |
|---|---|
| 18 | -0.11 |
| 19 | -0.06 |
| 20 | 0.00 |
| 21 | -0.32 |
| 22 | 0.05 |
| 23 | 1.00* |
| 24 | 0.00 |
| 25 | 0.25 |

Footnote: *This was based on only two data points.

For peer review only

BMJ Open

# Day to day variability of knee pain and the relationship with physical activity in people with knee osteoarthritis: an observational, feasibility study using consumer smartwatches

| | |
|---|---|
| Journal: | *BMJ Open* |
| Manuscript ID | bmjopen-2022-062801.R1 |
| Article Type: | Original research |
| Date Submitted by the Author: | 13-Oct-2022 |
| Complete List of Authors: | Vivekanantham, Arani ; The University of Manchester; Oxford University Hospitals NHS Trust, Department of Rheumatology<br>Selby, David; The University of Manchester<br>Lunt, Mark; The University of Manchester<br>Sergeant, Jamie; The University of Manchester<br>Parkes, Matthew; The University of Manchester<br>O'Neill, Terence; The University of Manchester, Arthritis Research UK Centre for Epidemiology; Salford Royal NHS Foundation Trust, Department of Rheumatology<br>Dixon, Will; The University of Manchester; Manchester Academic Health Science Centre |
| <b>Primary Subject Heading</b>: | Epidemiology |
| Secondary Subject Heading: | Rheumatology, Epidemiology, Sports and exercise medicine, Research methods, Patient-centred medicine |
| Keywords: | PAIN MANAGEMENT, EPIDEMIOLOGY, Knee < ORTHOPAEDIC & TRAUMA SURGERY |
| | |

SCHOLARONE™
Manuscripts

*I, the Submitting Author has the right to grant and does grant on behalf of all authors of the Work (as defined in the below author licence), an exclusive licence and/or a non-exclusive licence for contributions from authors who are: i) UK Crown employees; ii) where BMJ has agreed a CC-BY licence shall apply, and/or iii) in accordance with the terms applicable for US Federal Government officers or employees acting as part of their official duties; on a worldwide, perpetual, irrevocable, royalty-free basis to BMJ Publishing Group Ltd ("BMJ") its licensees and where the relevant Journal is co-owned by BMJ to the co-owners of the Journal, to publish the Work in this journal and any other BMJ products and to exploit all rights, as set out in our licence.*

*The Submitting Author accepts and understands that any supply made under these terms is made by BMJ to the Submitting Author unless you are acting as an employee on behalf of your employer or a postgraduate student of an affiliated institution which is paying any applicable article publishing charge ("APC") for Open Access articles. Where the Submitting Author wishes to make the Work available on an Open Access basis (and intends to pay the relevant APC), the terms of reuse of such Open Access shall be governed by a Creative Commons licence – details of these licences and which Creative Commons licence will apply to this Work are set out in our licence referred to above.*

*Other than as permitted in any relevant BMJ Author's Self Archiving Policies, I confirm this Work has not been accepted for publication elsewhere, is not being considered for publication elsewhere and does not duplicate material already published. I confirm all authors consent to publication of this Work and authorise the granting of this licence.*

For peer review only

# Day to day variability of knee pain and the relationship with physical activity in people with knee osteoarthritis: an observational, feasibility study using consumer smartwatches

Arani Vivekanantham [1, 2], David Selby [1], Mark Lunt [1], Jamie Sergeant [1], Matthew Parkes [1], Terence W O'Neill [1, 3, 4], William G Dixon [1, 3, 4]

[1] Centre for Epidemiology Versus Arthritis, University of Manchester, Manchester, UK.

[2] Department of Rheumatology, Nuffield Orthopaedic Centre, Oxford University Hospitals NHS Trust, Windmill Road, Oxford, UK.

[3] NIHR Manchester Biomedical Research Centre, Manchester University NHS Foundation Trust, Manchester Academic Health Science Centre, UK.

[4] Department of Rheumatology, Salford Royal NHS Foundation Trust, Salford, UK.

**Corresponding author**

Dr Arani Vivekanantham

Nuffield Department of Orthopaedics, Rheumatology and Musculoskeletal Sciences, Botnar Research Centre, Windmill Road, Oxford, OX3 7HD, UK.

arani.vivekanantham@ndorms.ox.ac.uk

**Email addresses for the co-authors:**

David Selby: david.selby@manchester.ac.uk, Mark Lunt: mark.lunt@manchester.ac.uk, Jamie Sergeant: jamie.sergeant@manchester.ac.uk, Matthew Parkes:

For peer review only

matthew.parkes@manchester.ac.uk, Terence W O'Neill: terence.o'neill@manchester.ac.uk,

William G Dixon: will.dixon@manchester.ac.uk

**Running title**

Pain and activity in knee osteoarthritis



For peer review only - http://bmjopen.bmj.com/site/about/guidelines.xhtml

## ABSTRACT

**Objective**: To assess the feasibility of using smartwatches in people with knee osteoarthritis (OA) to determine the day-to-day variability of pain and relationship between daily pain and step count.

**Design:** Observational, feasibility study.

**Setting:** In July 2017, the study was advertised in newspapers, magazines, and on social media. Participants had to be living/ willing to travel to Manchester. Recruitment was in September 2017 and data collection was completed in January 2018.

**Participants:** 26 participants age ≥50 years with self-diagnosed symptomatic knee OA were recruited.

**Outcome measures:** Participants were provided with a consumer cellular smartwatch with a bespoke app that triggered a series of daily questions including twice daily questions about level of knee pain and once monthly questions from the pain sub-scale of the Knee Injury and Osteoarthritis Outcome Score (KOOS) questionnaire. The smartwatch also recorded daily step counts.

**Results:** Of the 25 participants, 13 were male and their mean age was 65 years (standard deviation 8 years). The smartwatch app was successful in simultaneously assessing and recording data on knee pain and step count in real time. Knee pain was categorized into sustained high/ low or fluctuating levels, but there was considerable day to day variation within these categories. Levels of knee pain in general correlated with pain assessed by KOOS. Those


For peer review only



For peer review only - http://bmjopen.bmj.com/site/about/guidelines.xhtml

with sustained high/ low levels of pain had a similar daily step count average (mean 3754 (standard deviation (SD) 2524)/ 4307 (SD 2992)), but those with fluctuating pain had much lower step count levels (mean 2064 (SD 1716)).

**Conclusions:** Smartwatches can be used to assess pain and physical activity in knee OA. Larger studies may help inform a better understanding of causal links between physical activity patterns and pain. In time, this could inform development of personalized physical activity recommendations for people with knee OA.

## Keywords

Pain, knee osteoarthritis, mobile health (mHealth), patient-reported outcome, smartwatch.

**Strengths and limitations of this study**

- Our app was successful in assessing and recording data on patient reported symptoms and continuous sensor data simultaneously in real time.
- As this was a feasibility study, participant numbers were relatively small.
- Subjects volunteered for participation, and it is possible that selection factors and in particular an interest in smartwatches may have influenced engagement and reporting, limiting the generalization of the findings.
- Participants self-reported their knee OA; participants may have had varying severities of their knee OA (early disease to end-stage).



For peer review only - http://bmjopen.bmj.com/site/about/guidelines.xhtml

- The step count used was an estimate of the true step count levels, which was broken up by charging cycles meaning some activity may have been missed leading to measurement error; also, the total step count is a very rough measure of physical activity and misses patterns within the day.

## Introduction

Knee osteoarthritis (OA) affects one in eight people over the age of 60 years and pain is a cardinal symptom. Longitudinal studies suggest that the symptoms of knee OA typically follow relatively stable long-term trajectories (1)(2), however, this has been based on infrequently collected data. This is because clinical practice and most research studies capture pain reports at discrete time points, often months apart (3). We thus have a limited picture of daily pain trajectories; such data are important because variations in pain, and its inherent uncertainty and unpredictability, can impact significantly on patients' quality of life. It has more recently been proven that there is substantial day to day and within-day variability of pain in people with knee OA (4) (5). For example, in a prospective cohort study by Parry et al, they found that 23–32% of people with knee OA reported significant pain variability (4).

Pain in knee OA may be influenced by a range of other factors including levels of physical activity (6) (7). Like pain, physical activity assessment is also typically measured infrequently. Furthermore, it is often reliant on questionnaires which are subject to poor recall and poor precision. Understanding the relationship between pain and physical activity in knee OA has been limited by the ability to measure both variables simultaneously, accurately and in real

For peer review only



For peer review only - http://bmjopen.bmj.com/site/about/guidelines.xhtml

time. The use of objectively measured levels of physical activity have been recommended (8) (9), yet only three known longitudinal studies have assessed the relationship between pain and steps per day in people with knee OA using such measures. One used an accelerometer to measure step count (10) and the other two used a research-grade activity monitor (StepWatch; ActivPAL) (11) (12). However, in all three studies pain levels were measured at different time points, in other words not simultaneously, to the physical activity.

Consumer technology including smartphones and wearables provides opportunity to measure both patient reported outcomes (such as pain) and passively collected sensor data (such as physical activity) contemporaneously. Smartphone apps are increasingly being used to track symptoms including in rheumatic diseases (13). Although smartphones contain accelerometers allowing assessment of movement, they are not habitually carried by the user, and may thus misrepresent true patterns of activity (14). Dedicated wearable devices like fitness trackers, often wrist-worn, can continuously track movement, but rarely support concurrent data entry.

In this observational feasibility study, called 'Knee OsteoArthritis: Linking Activity and Pain' (KOALAP), we developed a cellular smartwatch app to record and assess pain in real time whilst also collecting raw and processed sensor data allowing contemporaneous capture of physical activity. The primary aim of the feasibility study was to test the feasibility, acceptability, and ongoing engagement with smartwatch data collection for research (15). In this analysis of data

For peer review only



For peer review only - http://bmjopen.bmj.com/site/about/guidelines.xhtml

from the KOALAP study, we sought to examine patterns of pain and physical activity in people aged over 50 living with OA. Specific objectives were to:

1. To describe the day to day and within-day variability of pain intensity in people with symptomatic knee OA.

2. To assess how daily pain scores compare to pain assessed using monthly Knee Injury and Osteoarthritis Outcome Score (KOOS) pain domain scores.

3. To identify clusters of people with similar longitudinal pain scores.

4. To assess whether daily step count levels, assessed using the sensor data, are correlated with daily pain levels.

## Methods

### Study design and sample

KOALAP was a longitudinal, observational feasibility smartwatch study in people with self-reported symptomatic knee OA. The full protocol for the KOALAP study has been published elsewhere (15) and there were no deviations from this protocol. In July 2017, the study was advertised in local newspapers and magazines and via social media channels. Participants had to be aged 50 years or over, have a self-report diagnosis of knee OA and be living in the Greater Manchester area or willing to travel to Manchester. Interested participants were asked to contact the study team, after which they were sent a patient information sheet and an invitation to one of four enrollment events. Participants were recruited to the study in



For peer review only - http://bmjopen.bmj.com/site/about/guidelines.xhtml

September 2017 and data collection was completed in January 2018. The participants provided written informed consent for their participation in the study. The study involves human participants and was approved by the University of Manchester Research Ethics Committee (#0165).

**Sample size**

A minimum sample size of 20 participants was required based on expected attrition in order to describe patterns of longitudinal engagement, the primary aim of this feasibility study (15) (16).

**Data collection**

At the enrollment event participants self-reported their age, gender, height, and weight. They were then provided with a consumer cellular smartwatch (Huawei Watch 2) with a bespoke app that collected i) patient-reported outcomes via questionnaires and ii) continuous watch sensor data. The participants were instructed to wear the watch from waking until going to bed and to answer the watch questions when prompted within a specific time window, illustrated in Table 1. All data were collected daily for 90 consecutive days.

**Table 1: Summary of the frequency, trigger times and completion window times of the smartwatch questions**

| Question | Frequency | Trigger time | Window |
|---|---|---|---|



For peer review only - http://bmjopen.bmj.com/site/about/guidelines.xhtml

| | | | |
|---|---|---|---|
| Level of knee pain | Twice daily | 12.22 and 18.22 | 4 hours |
| 26 items from the Knee Injury and Osteoarthritis Outcome Score (KOOS) questionnaire | Monthly | Days 14, 44, 74 from start point | 1 week |

For the twice daily 'level of knee pain' questions participants were asked to record their level of knee pain on a 0-10 numeric rating scale (17). To reduce responder burden, the KOALAP app used a subset of 26 items from the KOOS questionnaire in the app. This included the sub-scales on 'pain' and 'function, activities of daily living' (items P1-P9 and A1-A17 respectively illustrated in the Appendix, Table A1). These sub-scales were felt to contain the items most directly relevant to pain and function (18). For this study, we decided to only include data from the 'pain' sub-scale (i.e., questions P2- P9 only). Participants were asked to score their responses on the following five-point scale: none (0), mild (1), moderate (2), severe (3) and extreme (4). We then took the sum of these item scores for each participant to give them a score for each month. We then scaled the score to a percentage, given the maximum possible score was 32, and divided by 10 to give us a score in the range between 1-10. We did not impute any missing data.

**Statistical methods**



For peer review only - http://bmjopen.bmj.com/site/about/guidelines.xhtml

160 Descriptive statistics were used to describe participant characteristics including mean and standard deviation (SD) or median and interquartile range (IQR) for continuous data, and frequency (%) for categorical variables. Body mass index (BMI) was calculated from participants' height (meters) divided by weight (kilograms) squared.

*Questionnaire data*

We plotted twice daily pain scores over the 90-day period on individual graphs for each of the participants. On assessing data quality descriptively prior to the main analysis, we believed certain scores of 0 may have been submitted by accident. This was due to the user interface of the smartwatch app having a default value of zero and thus occasionally generating zero-values as an error in data input. Given this error in the data input, all pain scores of zero (of which there were 212 in total) were treated as missing values. Individual daily pain scores (numerical rating scale 1-10) were re-plotted, then visually reviewed for distribution patterns. The difference between early evening and lunchtime pain scores for each day of the 90-day period was calculated to assess within-day variability in level of knee pain. Monthly KOOS pain scores for each participant were plotted on top of the day-to-day pain scores.

The mean and variance of the lunchtime and early evening pain scores were calculated for each participant over the whole study period, giving an indication of the average level of pain and its variability within each participant's time series. These participant-level summary statistics were passed into a k-means algorithm (19). From the plotted graphs of trajectories of daily pain scores for each of the participants, three broad patterns of pain were identified: sustained high



For peer review only - http://bmjopen.bmj.com/site/about/guidelines.xhtml

levels of pain, sustained low levels of pain and fluctuating levels of pain. Based on this clusters (k=3) were chosen for the algorithm; this grouped the participants into the three respective pain level groups. This was done separately for both the lunchtime and early evening pain; as the patterns of pain were similar, we decided to present only the lunchtime pain scores. In line with previous literature by Hawker and colleagues who have written extensively about the idea of OA causing two types of pain: intermittent (variable) and constant (stable) (20), we also explored a 2-class model (based on variance only) which included constant (stable) pain and intermittent (variable) pain.

*Sensor data*

Physical activity levels were assessed as total steps per day, measured by the app. The step counts were estimated from the accelerometry data using a proprietary algorithm. For our analysis, only estimated step counts over each calendar day were required. However, the data from the smartwatch were not recorded as daily step counts; but rather a monotonically increasing number sequence that re-sets at irregular intervals, corresponding to times when the smartwatch was re-charged. To convert these data into daily step counts, we calculated the consecutive differences (i.e., the increases from one data point to the next recorded data point, for that person) of the raw counts (i.e., counts as recorded by the smartwatch) for each user. Where the *raw* differences were negative (i.e., when the raw count has re-set indicating the internal stepometer has re-set) or the time lag between consecutive data points were more than one day, the difference was re-set to zero. Resetting differences over more than one day to zero is important, because it is not known if the step count after such a gap is due to the

For peer review only



For peer review only - http://bmjopen.bmj.com/site/about/guidelines.xhtml

accumulation of multiple days, or whether they were all from that single day. Therefore, it avoids spuriously high step counts for certain days. The sum of this modified sequence was then computed for each calendar date for each participant, giving the estimated daily step counts. We looked then at the mean and variance of step counts by pain cluster group. We looked also at the correlation between same day morning pain scores and step counts by participant using Pearson correlation.

All the statistical analyses were carried out using R version 3.6.0 (21). The R packages dplyr and tidyr (22) were used for data manipulation and the package ggplot2 (23) was used to produce the graphs. The R code used for analysis is included as a supplementary file.

**Missing data**

We did not impute missing data as missing data may have not been missing at random. Patient may have stopped responding to the study either because they felt they do not have any symptoms worth reporting, or because they are in so much discomfort that using the app may have been a burden.

**Patient and public involvement**

None.



For peer review only - http://bmjopen.bmj.com/site/about/guidelines.xhtml

## Results

### Participant characteristics

A total of 26 participants were recruited. One participant left the study early and was excluded from the analysis. The mean age of the participants was 65 years, (SD= 8 years). Of the 25 participants, just over half (n=13) were male. The median (interquartile range) BMI was 27 (25, 35) $kg/m^2$.

### Daily knee pain

The twice daily questions were completed on around half of the participant-days (16). In total, there were 2756 pain reports (out of a potential of 4680 pain reports), giving an average completion rate of approximately 59% for these questions. Of these, 78 were zero values which were then excluded from the analysis given the concern about false entries (see methods section and Figure A1 in the Appendix). Day-to-day patterns of knee pain, assessed at 12.22pm, for each of the 25 participants over the three-month study period is presented in Figure 1. The data show that there is a considerable amount of variation in the day-to-day levels of knee pain in the participants. Some participants (for example participant IDs 3 and 12) had sustained high levels of pain, some participants (for example participant ID 18) had sustained low levels of pain, while others (for example, participant IDs 1, 2, 9, 11) had more fluctuating pain levels.

For the majority of participants (n=20) there was no overall difference between their daily 12.22pm and 18.22pm pain scores (median difference of zero (IQR 1)). In the remaining five



For peer review only - http://bmjopen.bmj.com/site/about/guidelines.xhtml

participants, four reported worse pain in the early evening (participant IDs 9, 16, 24, 25) as illustrated graphically in Figure 2, which shows a Bland-Altman plot of self-reported daily scores for overall early evening versus overall lunchtime pain, by participant. For most participants, the mean difference is near zero. There are some users for whom early evening pain scores is slightly higher (for example, participant ID 9).

As there was no difference in daily pain scores between the two time points, in the future analysis included in this manuscript we focus only on the lunchtime pain scores.

**Monthly KOOS pain scores**

The monthly KOOS pain scores (where available) for each participant were plotted alongside their day-to-day levels of pain, as red dots, in Figure 1. It is not possible to directly compare the KOOS pain score and the daily level of pain, however, there were some interesting observations seen. For example, the KOOS pain scores were sometimes lower than that suggested by the daily pain scores (for example in participant IDs 3 and 10) and sometimes the KOOS pain scores were higher than the daily pain scores (for example in participant IDs 4 and 18). For most of the participants the degree of variability in the KOOS pain scores within an individual was less marked than the day-to-day variability in pain though apart from perhaps one or two participants (for example, participant ID 3) tended to follow the broad pattern of daily pain over the 3 months.



For peer review only - http://bmjopen.bmj.com/site/about/guidelines.xhtml

**Cluster analysis**

The mean and variance of the lunchtime (as well as early evening) pain scores for each of the participants are shown in the Appendix (in Table A2). Using the k algorithm and including k-3 subjects were characterized into clusters (see Figure 3). For each participant ID we calculated group-wise summary statistics: namely the sample mean of the morning pain and respectively of the afternoon pain scores and the sample variances of the same. Hence each patient is represented as a vector of four components: the mean and standard deviation of morning pain and of afternoon pain. These vectors are passed into the "kmeans()" function from the base R stats package, with k = 3 with 10 random restarts and the default Hartigan–Wong algorithm selected. The clusters described are the output from this function. On review of the lunchtime pain patterns (as shown in Figure 1), it suggested three broad clusters of pain: high, low, and fluctuating levels of pain. We repeated the clustering having removed participants who dropped out in the first half of the study as a sensitivity analysis and found the same result.

As mentioned in the methods section, we also explored a 2-class model (based on variance only) which included constant (stable) pain and intermittent (variable) pain. Our findings of this are illustrated in Figure A2 in the Appendix. We decided to focus on the 3-cluster model in this paper and present the above methods in the main paper.

**Physical activity/step count data**



For peer review only - http://bmjopen.bmj.com/site/about/guidelines.xhtml

The estimated daily step count levels for each of the 25 participants over the three-month period is shown in Figure 4. The figure highlights the variability in step counts with some participants having little variability (for example, participant IDs 11 and 12), others having high variability (for example, participant IDs 24 and 25) and one (participant ID 4) having two very distinct patterns of activity.

To explore whether the differences in step count levels between the participants was linked to their daily pain levels, we calculated the mean (and standard deviation) step count per day for the three clusters, as shown in Table 2. We found that all clusters had low levels of physical activity, especially the fluctuating pain levels cluster. Those with sustained high or sustained low pain levels had a similar step count level, however, those with fluctuating pain levels (i.e., unpredictable levels of pain) had much lower step count levels.

For peer review only

**Table 2: Mean and standard deviation of daily step count by pain cluster**

| Pain cluster | | |
|---|---|---|
| | **Mean** | **Standard deviation** |
| Sustained high pain levels | 3754 | 2524 |
| Sustained low pain levels | 4307 | 2992 |
| Fluctuating pain levels | 2064 | 1716 |

In an individual analysis, for the majority of participants (n=17) the correlation coefficients were positive indicating that increased step count was linked with increasing pain however the correlation coefficients were small, confidence intervals were wide and embraced unity, as illustrated in Table A3 in the Appendix. In some participants who had sustained high lunchtime pain scores, step count levels were low (for example, participant ID 3 and 12). However, other participants who had sustained low lunchtime pain scores, also had low step count levels (for example, participant ID 15). In those participants who had fluctuating pain scores (ranging from low to high), some had fluctuating step count levels (ranging from 0 steps to > 20,000) (for example, participant IDs 1, 24, 25) whereas others had sustained low step counts (for example, participant IDs 2, 9 ,11) irrespective of high or low pain scores, as shown in Figure 5.

## Discussion



For peer review only - http://bmjopen.bmj.com/site/about/guidelines.xhtml

In our study, among people with knee OA we found that there was little within day but significant between day variability in pain. We found that when we categorised pain into three groups, patients separated into intermittent (variable) and constant, either sustained high or low levels of pain, supporting what is already known (7) (24) . We also found that there was no difference in pain scores between the lunchtime and early evening scores in most of our participants though it may be that the app timings (notifications at 12.22 and 18.22) were not truly representative of the maximal possible range of pain from morning to evening. Our study also found that participants with seemingly stable monthly KOOS pain scores had a lot of day-to-day variability in pain levels in between their monthly scores.

Our study also uniquely found that those with either sustained high or low levels of pain have a similar step count average but those with fluctuating pain have much lower levels of activity. This may indicate that fluctuating pain (i.e., pain that is unpredictable/ always changing) may be more troublesome to people living with knee OA than those with, for example, sustained high levels of pain. Our finding that low levels of pain was not necessarily associated with higher step count levels highlights that there may be other factors related to low physical activity levels in people with knee OA (not just pain). For example, White et al found that people with depressive symptoms at baseline walked fewer steps per day (11). It is also important to note the possible bidirectional nature of pain and physical activity, whereby low pain may enable greater activity, but also where increased activity may precipitate higher pain. Our analysis of



For peer review only - http://bmjopen.bmj.com/site/about/guidelines.xhtml

this feasibility study did not examine this in detail, yet it provides evidence this might be possible in the future given the ability to collect time series data.

Studies to date remain ambiguous regarding the link between the pain in knee OA and physical activity, with some studies finding a positive association (6) and some studies no association (10). These differences in findings may in part be due to the different methods used to measure physical activity and the timings when the level of pain was assessed. For example, in a study by Parry et al, paper diaries were used to collect information on daily pain in people with knee OA, to assess the impact of specific physical exposures on increases in daily pain levels (6). However, as these paper diaries were completed retrospectively, they may have been subject to errors of inaccurate recall. With the use of this novel method of using consumer devices to measure symptoms and step count concurrently, it may help provide a more accurate assessment of these associations in the future.

There are, however, also several limitations to be considered when interpreting the findings from this study. This was a feasibility study and so the participant numbers were relatively small and biased with non-random missingness, so any conclusions drawn may not necessarily generalize to all future users of such an app or all people with knee osteoarthritis. Subjects volunteered for participation, and it is possible that selection factors and in particular an interest in smartwatches may have influenced engagement and reporting, limiting the generalization of the findings. Participants self-reported their knee OA; this diagnosis was not

For peer review only



For peer review only - http://bmjopen.bmj.com/site/about/guidelines.xhtml

confirmed with their medical records, we do not know if they are receiving any treatments, we did not have information on disease duration and thus participants have had varying severities of their knee OA (early disease to end-stage). Moreover, due to the user interface of the smartwatch app having a default value of zero and thus occasionally generating zero-values as an error in data input, all zero values were treated as missing values to avoid 'false' zeros; we recognize that in doing this we may have also removed some 'true' zeros.

There are also several limitations related to the step-count. The watch measured accelerometry data and a proprietary algorithm estimated the number of steps from these data. However, in a study that compared commercially available activity tracking devices for step count accuracy no significant differences were found among the devices (25). The step count used was an estimate of the true step count levels, which was broken up by charging cycles- some activity may have been missed, leading to measurement error. Also, the total step count is a very rough measure of physical activity and misses patterns within the day (25). Moreover, the qualitative details, such as whether the walking was slow/ fast (which have stronger associations with pain), was missed. We do, however, have the raw accelerometer data and will conduct future analysis to help us to better understand the relationship between pain and more granular patterns of physical activity. Our analysis describes three clusters of longitudinal pain patterns and their physical activity. The descriptive analysis was exploratory rather than intending to formally compare these groups given the low sample size. This could be done in future studies.



For peer review only - http://bmjopen.bmj.com/site/about/guidelines.xhtml

It is well known that physical activity and exercise play a critical role in the conservative management of knee OA (26), however physical activity-based exposures may also trigger acute flares. The findings from this study support the notion that a one size fits all management of pain and physical activity in knee OA may not be effective and that a more personalized approach may be needed. In the future, the use of consumer devices may aid this personalized approach as it will help to identify the optimal level of physical activity for each patient before it triggers an exacerbation of pain. This opens the opportunity for personalised (digital) coaching, where patients might be guided to do a level of physical activity that is appropriate for them, encouraging activity levels up to, but not exceeding, a level that exacerbates pain. In summary, this feasibility study has shown that the use of the consumer cellular smartwatch app was successful in assessing and recording data on patient reported outcomes and continuous sensor data simultaneously in real time. This data allowed us to highlight the substantial variability in knee pain due to OA and its links with physical activity. Larger studies are required to confirm our findings and allow exploration of the complex bidirectional relationships between activity and pain. This method of data capture should help to develop personalized physical activity recommendations for people with knee OA in the future, based on what their optimal level of physical activity is in relation to their pain.

For peer review only



For peer review only - http://bmjopen.bmj.com/site/about/guidelines.xhtml

## Contributors

WGD, TON, JS, and MP were involved in the conception, design, and data collection for the study. DS and AV were involved in the data analysis and all authors (AV, DS, WGD, TON, JS, MP and ML) were involved in the interpretation of the results. AV drafted the article with critical revision from all authors (AV, DS, WGD, TON, JS, MP and ML). All authors (AV, DS, WGD, TON, JS, MP and ML) approved the final version of the article to be published.

## Acknowledgements

This project has been possible through collaboration with the Google Fit Research team at Google UK. The Google team has collaboratively built the KOALAP app for self-reported data collection and the system for collecting and transmitting sensor data.

## Funding

The study was funded by Versus Arthritis (grants 21225 and 21755) and supported by the NIHR Manchester Biomedical Research Centre (grant number N/A). AV is supported by a National Institute for Health and Research (NIHR) funded Academic Clinical Fellowship (grant number N/A). The views expressed in this publication are those of the author(s) and not necessarily those of the NHS, the NIHR or the Department of Health.

## Competing interests

WGD has received consultancy fees from Google and Abbvie.

## Data availability statement

No additional data available



For peer review only - http://bmjopen.bmj.com/site/about/guidelines.xhtml

## Ethics approval

The study involves human participants and was approved by the University of Manchester Research Ethics Committee (#0165).

## References

1. Soni A, Kiran A, Hart DJ, Leyland KM, Goulston L, Cooper C, et al. Prevalence of reported knee pain over twelve years in a community-based cohort. Arthritis Rheum. 2012;64(4):1145-52.

2. Cedraschi C, Delézay S, Marty M, Berenbaum F, Bouhassira D, Henrotin Y, et al. "Let's talk about OA pain": a qualitative analysis of the perceptions of people suffering from OA. Towards the development of a specific pain OA-Related questionnaire, the Osteoarthritis Symptom Inventory Scale (OASIS). PLoS One. 2013;8(11):e79988.

3. Allen KD, Coffman CJ, Golightly YM, Stechuchak KM, Keefe FJ. Daily pain variations among patients with hand, hip, and knee osteoarthritis. Osteoarthritis Cartilage. 2009;17(10):1275-82.

4. Schneider S, Junghaenel DU, Keefe FJ, Schwartz JE, Stone AA, Broderick JE. Individual differences in the day-to-day variability of pain, fatigue, and well-being in patients with rheumatic disease: associations with psychological variables. Pain. 2012;153(4):813-22.

5. Parry E, Ogollah R, Peat G. Significant pain variability in persons with, or at high risk of, knee osteoarthritis: preliminary investigation based on secondary analysis of cohort data. BMC Musculoskelet Disord. 2017;18(1):80.

6. Parry E, Ogollah R, Peat G. 'Acute flare-ups' in patients with, or at high risk of, knee osteoarthritis: a daily diary study with case-crossover analysis. Osteoarthritis Cartilage. 2019;27(8):1124-8.

For peer review only



For peer review only - http://bmjopen.bmj.com/site/about/guidelines.xhtml

7. Thomas MJ, Butler-Walley S, Rathod-Mistry T, Mayson Z, Parry EL, Pope C, et al. Acute flares of knee osteoarthritis in primary care: a feasibility and pilot case-crossover study. Pilot Feasibility Stud. 2018;4:167.

8. Prince SA, Adamo KB, Hamel ME, Hardt J, Connor Gorber S, Tremblay M. A comparison of direct versus self-report measures for assessing physical activity in adults: a systematic review. Int J Behav Nutr Phys Act. 2008;5:56.

9. Verlaan L, Bolink SA, Van Laarhoven SN, Lipperts M, Heyligers IC, Grimm B, et al. Accelerometer-based Physical Activity Monitoring in Patients with Knee Osteoarthritis: Objective and Ambulatory Assessment of Actual Physical Activity During Daily Life Circumstances. Open Biomed Eng J. 2015;9:157-63.

10. Brisson NM, Gatti AA, Maly MR. Association of Pain and Steps Per Day in Persons With Mild-to-Moderate, Symptomatic Knee Osteoarthritis: A Mixed-Effects Models Analysis of Multiple Measurements Over Three Years. Arthritis Care Res (Hoboken). 2020;72(1):114-21.

11. White DK, Tudor-Locke C, Zhang Y, Niu J, Felson DT, Gross KD, et al. Prospective change in daily walking over 2 years in older adults with or at risk of knee osteoarthritis: the MOST study. Osteoarthritis Cartilage. 2016;24(2):246-53.

12. Matthew J. Parkes RKJ, Suzanne C. Carter, Anmin Liu, Michael J. Callaghan, David T. Felson. Change in pain and its relation to change in activity in osteoarthritis. Osteoarthritis and Cartilage Open. 2020;2(3).

13. Dixon WG, Michaud K. Using technology to support clinical care and research in rheumatoid arthritis. Curr Opin Rheumatol. 2018;30(3):276-81.

14. Silva AG, Simões P, Queirós A, Rodrigues M, Rocha NP. Mobile Apps to Quantify Aspects of Physical Activity: a Systematic Review on its Reliability and Validity. J Med Syst. 2020;44(2):51.

For peer review only



For peer review only - http://bmjopen.bmj.com/site/about/guidelines.xhtml

15. Beukenhorst AL, Parkes MJ, Cook L, Barnard R, van der Veer SN, Little MA, et al. Collecting Symptoms and Sensor Data With Consumer Smartwatches (the Knee OsteoArthritis, Linking Activity and Pain Study): Protocol for a Longitudinal, Observational Feasibility Study. JMIR Res Protoc. 2019;8(1):e10238.

16. Beukenhorst AL, Howells K, Cook L, McBeth J, O'Neill TW, Parkes MJ, et al. Engagement and Participant Experiences With Consumer Smartwatches for Health Research: Longitudinal, Observational Feasibility Study. JMIR Mhealth Uhealth. 2020;8(1):e14368.

17. Turk DC, Dworkin RH, Burke LB, Gershon R, Rothman M, Scott J, et al. Developing patient-reported outcome measures for pain clinical trials: IMMPACT recommendations. Pain. 2006;125(3):208-15.

18. Roos EM, Roos HP, Lohmander LS, Ekdahl C, Beynnon BD. Knee Injury and Osteoarthritis Outcome Score (KOOS)--development of a self-administered outcome measure. J Orthop Sports Phys Ther. 1998;28(2):88-96.

19. Hartigan JA WMAA. A K-Means Clustering Algorithm. Journal of the Royal Statistical Society. 1979;28(1):100.

20. Carlesso LC, Hawker GA, Torner J, Lewis CE, Nevitt M, Neogi T. Association of Intermittent and Constant Knee Pain Patterns With Knee Pain Severity and With Radiographic Knee Osteoarthritis Duration and Severity. Arthritis Care Res (Hoboken). 2021;73(6):788-93.

21. Team RC. R: A Language and Environment for Statistical Computing. 2019 [

22. Wickham H HL. Tidyr: Tidy Messy Data. 2020.

23. H W. Ggplot2: Elegant Graphics for Data Analysis. 216.

24. Thomas MJ, Rathod-Mistry T, Harper S, Parry EL, Pope C, Neogi T, et al. Acute Flares of Knee Osteoarthritis (the ACT-FLARE Study): Protocol for a Web-Based Case-Crossover Study in Community-Dwelling Adults. JMIR Res Protoc. 2019;8(4):e13428.

For peer review only



For peer review only - http://bmjopen.bmj.com/site/about/guidelines.xhtml

25. Gaz DV, Rieck TM, Peterson NW, Ferguson JA, Schroeder DR, Dunfee HA, et al. Determining the Validity and Accuracy of Multiple Activity-Tracking Devices in Controlled and Free-Walking Conditions. Am J Health Promot. 2018;32(8):1671-8.

26. Uthman OA, van der Windt DA, Jordan JL, Dziedzic KS, Healey EL, Peat GM, et al. Exercise for lower limb osteoarthritis: systematic review incorporating trial sequential analysis and network meta-analysis. Bmj. 2013;347:f5555.

## FIGURE TITLES AND LEGENDS

**Figure 1: Daily pain scores (assessed at 12.22pm) and monthly Knee Injury and Osteoarthritis Outcome Score (KOOS) scores (represented by the red dots), by participant**

**Figure 2: Bland-Altman plot of self-reported daily scores for overall early evening versus overall lunchtime pain, by participant**

The black line is the mean difference, the dashed lines are +/- 1.96 * SD (difference).

**Figure 3: Variance of lunchtime pain by mean pain score, with each point representing a pain cluster**

The red circles (cluster 1) represent users with sustained high levels of pain, the blue squares (cluster 3) represent those with sustained low levels of pain, and the green triangles (cluster 2) represent those with fluctuating pain levels.

**Figure 4: Daily step count levels by participant, over the three-month study period**

**Figure 5: Jittered scatter plot of daily step count level against overall lunchtime pain, by**



For peer review only - http://bmjopen.bmj.com/site/about/guidelines.xhtml

**participant**

For peer review only


For peer review only - http://bmjopen.bmj.com/site/about/guidelines.xhtml

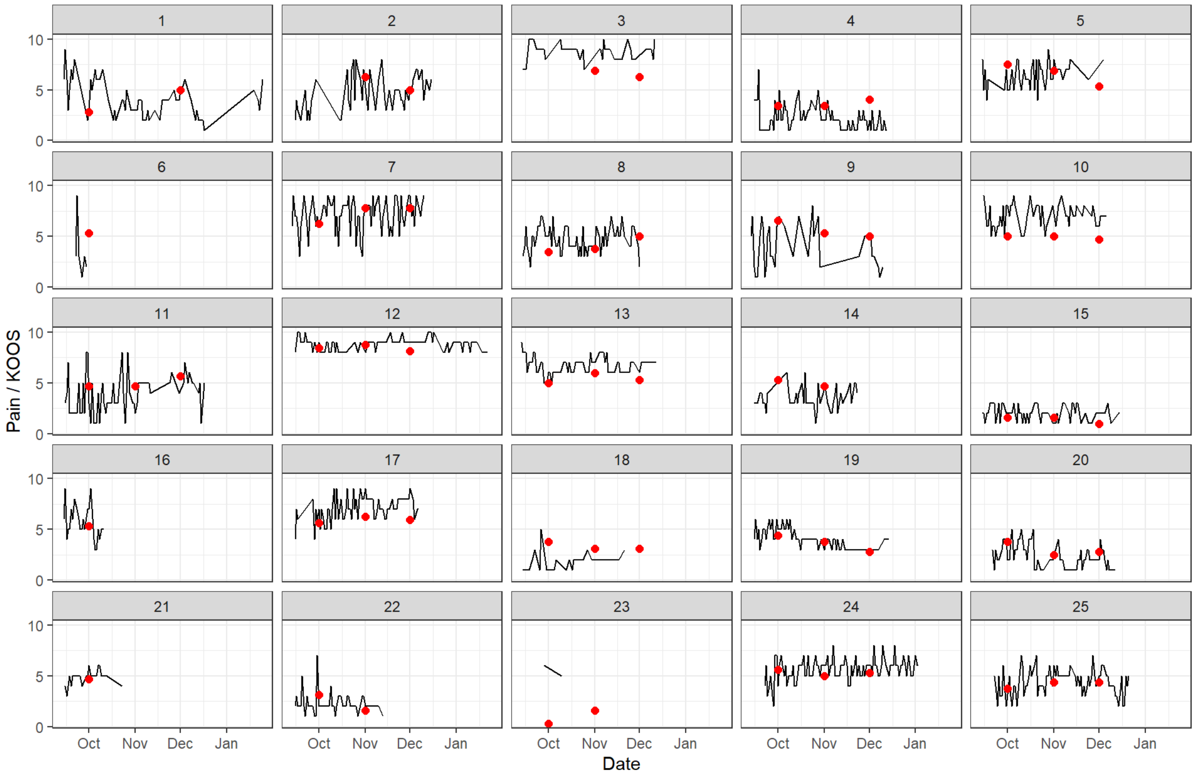


Figure 1: Daily pain scores (assessed at 12.22pm) and monthly Knee Injury and Osteoarthritis Outcome Score (KOOS) scores, represented by the red dots, by participant ID.

For peer review only

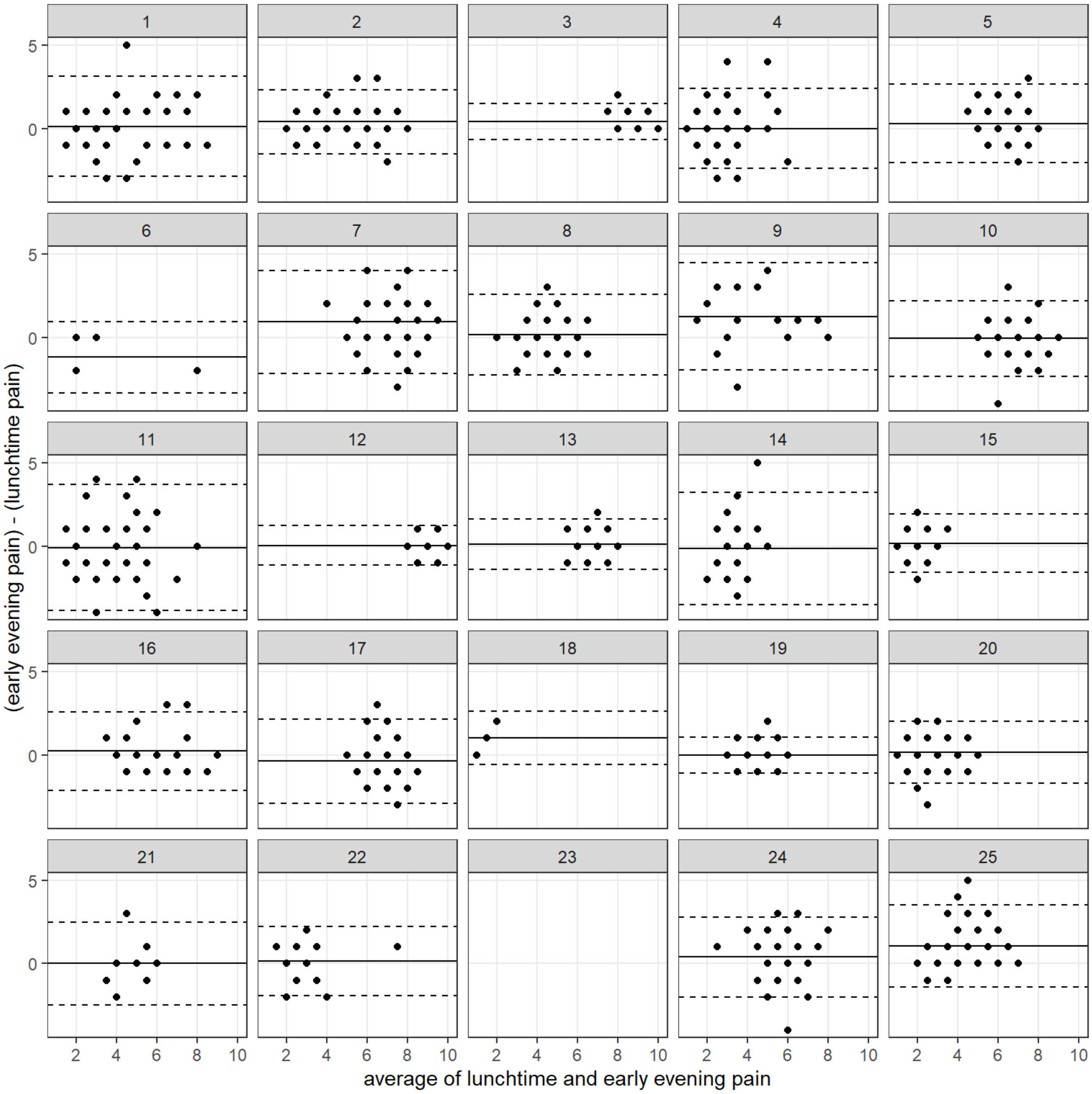


Figure 2: Bland-Altman plot of self-reported daily scores for overall early evening versus overall lunchtime pain, by participant. The black line is the mean difference, the dashed lines are +/- 1.96 * SD (difference).

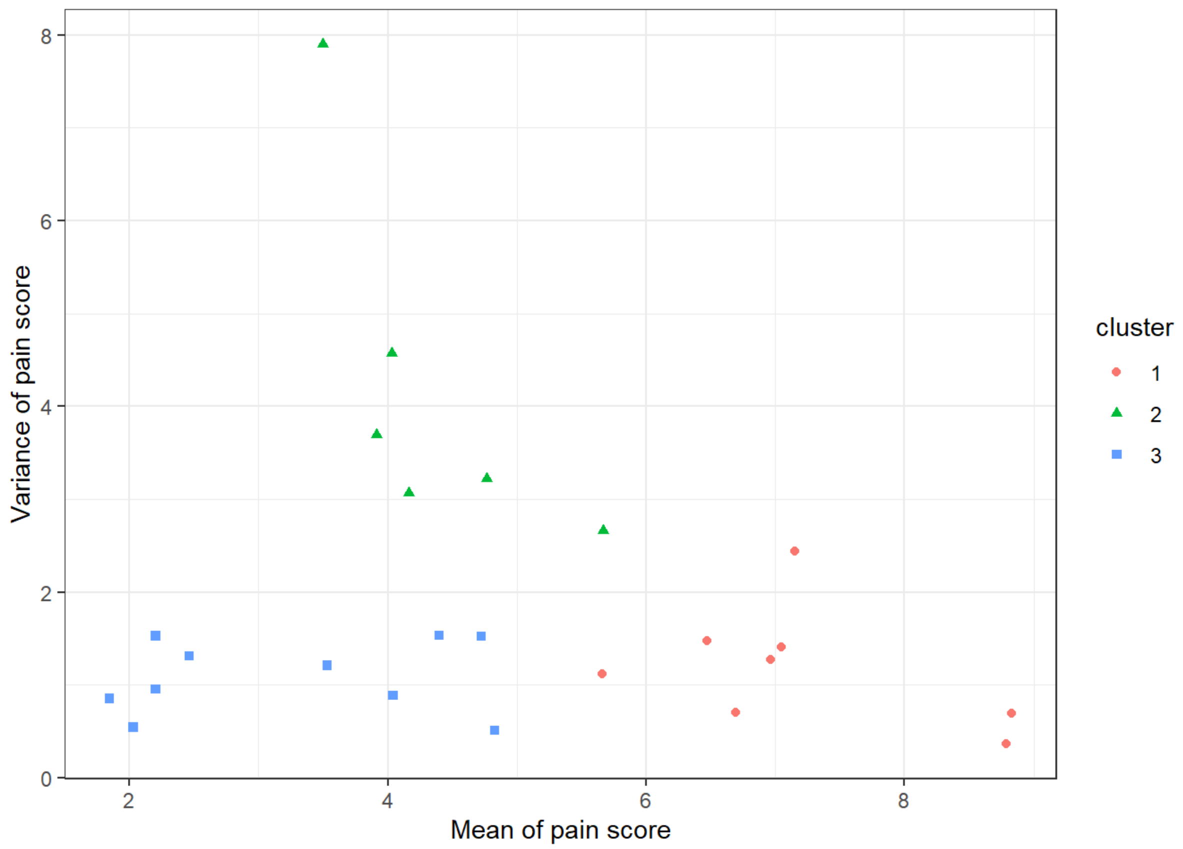


Figure 3: Variance of lunchtime pain by mean pain score with each point representing a pain cluster. The red circles (cluster 1) represent users with sustained high levels of pain, the blue squares (cluster 3) represent those with sustained low levels of pain, and the green triangles (cluster 2) represent those with fluctuating pain levels.

review only

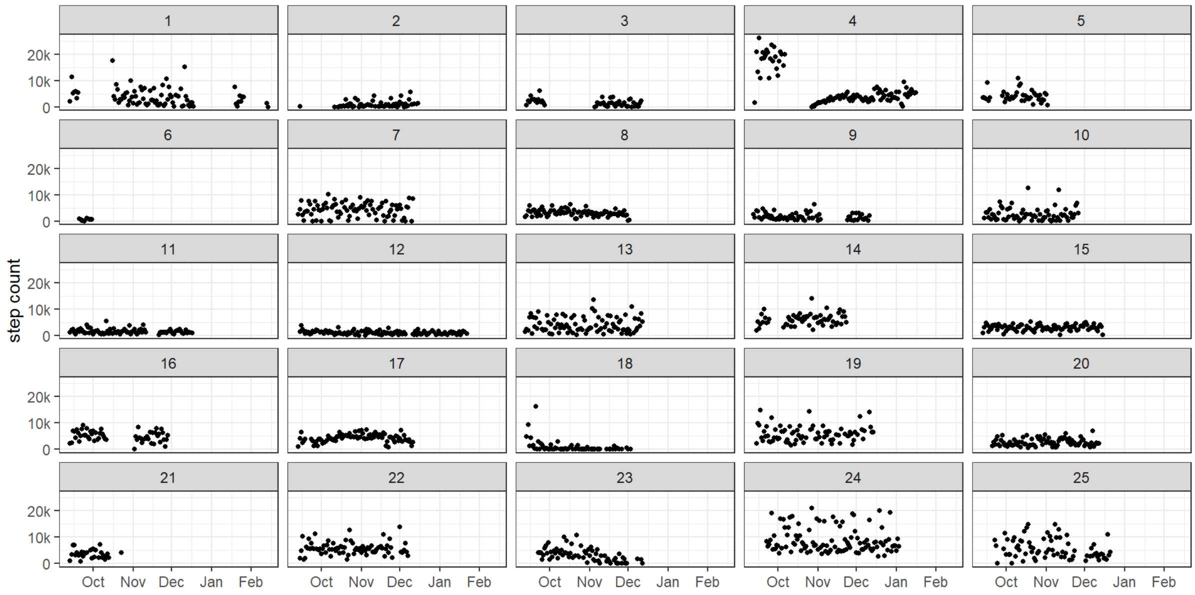


Figure 4: Daily step count levels by participant ID over the three-month study period.

For peer review only

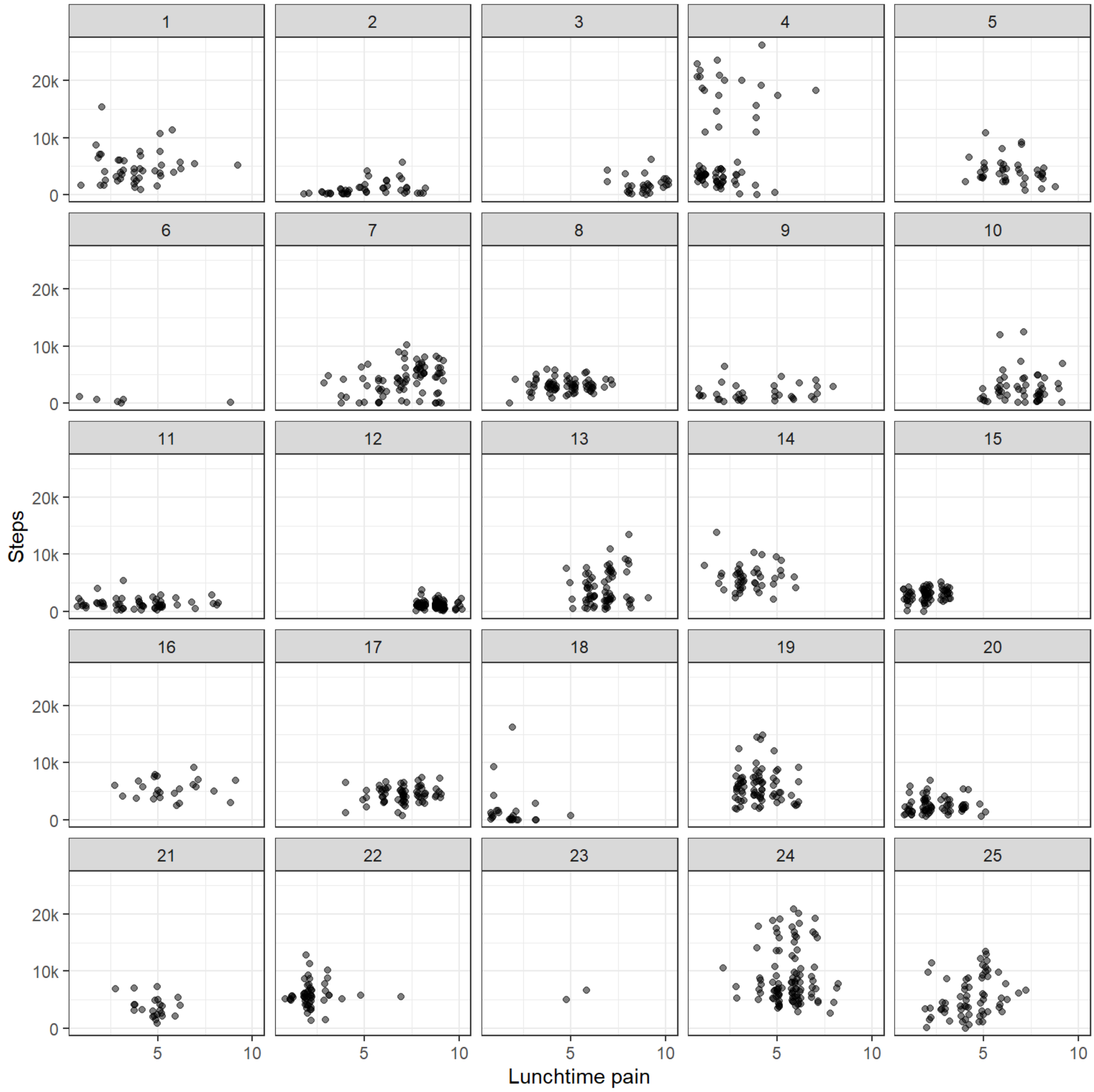


Figure 5: Jittered scatter plot of daily step count level against overall lunchtime pain, by participant.

Table A1: 17 questions from the Knee Injury and Osteoarthritis Outcome Score (KOOS) questionnaire that was used in this study.

| Sub-scale | Component | Response |
|---|---|---|
| **PAIN** | | |
| P1 | How often do you experience knee pain? | None/ Mild/ Moderate/ Severe/ Extreme |
| *What amount of knee pain have you experienced the last week during the following activities?* | | |
| P2 | Twisting/pivoting on your knee | None/ Mild/ Moderate/ Severe/ Extreme |
| P3 | Straightening knee fully | |
| P4 | Bending knee fully | |
| P5 | Walking on flat surface | |
| P6 | Going up or down stairs | |
| P7 | At night while in bed | |
| P8 | Sitting or lying | |
| P9 | Standing upright | |
| **FUNCTION, DAILY LIVING** *The following questions concern your physical function. By this we mean your ability to move around and to look after yourself. For each of the following activities please indicate the degree of difficulty you have experienced in the last week due to your knee.* | | |
| A1 | Descending stairs | None/ Mild/ Moderate/ Severe/ Extreme |
| A2 | Ascending stairs | |

For peer review only

| | | |
|---|---|---|
| *For each of the following activities please indicate the degree of difficulty you have experienced in the last week due to your knee.* | | |
| A3 | Rising from sitting | None/ Mild/ Moderate/ Severe/ Extreme |
| A4 | Standing | |
| A5 | Bending to floor/pick up an object | |
| A6 | Walking on flat surface | |
| A7 | Getting in/out of car | |
| A8 | Going shopping | |
| A9 | Putting on socks/stockings | |
| A10 | Rising from bed | |
| A11 | Taking off socks/stockings | |
| A12 | Lying in bed (turning over, maintaining knee position) | |
| A13 | Getting in/out of bath | |
| A14 | Sitting | |
| A15 | Getting on/off toilet | |
| *For each of the following activities please indicate the degree of difficulty you have experienced in the last week due to your knee* | | |
| A16 | Heavy domestic duties (moving heavy boxes, scrubbing floors, etc) | None/ Mild/ Moderate/ Severe/ Extreme |
| A17 | Light domestic duties (cooking, dusting, etc) | |

3



For peer review only - http://bmjopen.bmj.com/site/about/guidelines.xhtml

Table A2: Mean and variances of pain score by Participant ID

| Participant ID | Mean lunchtime pain level | Standard deviation lunchtime pain level | Mean early evening pain level | Standard deviation early evening pain level |
|---|---|---|---|---|
| 1 | 4.2 | 1.8 | 4.3 | 2.1 |
| 2 | 4.8 | 1.8 | 5.2 | 1.9 |
| 3 | 8.8 | 0.8 | 9.2 | 0.6 |
| 4 | 2.2 | 1.2 | 2.3 | 1.4 |
| 5 | 6.5 | 1.2 | 6.7 | 0.9 |
| 6 | 3.5 | 2.8 | 2.8 | 2.5 |
| 7 | 7.2 | 1.6 | 8.1 | 1.4 |
| 8 | 4.7 | 1.2 | 4.5 | 1.3 |
| 9 | 4.0 | 2.1 | 5.0 | 2.0 |
| 10 | 7.0 | 1.1 | 6.8 | 1.1 |
| 11 | 3.9 | 1.9 | 4.2 | 2.1 |
| 12 | 8.8 | 0.6 | 8.9 | 0.5 |
| 13 | 6.7 | 0.8 | 6.7 | 0.8 |

For peer review only

| | | | | |
|---|---|---|---|---|
| 14 | 3.5 | 1.1 | 3.4 | 1.2 |
| 15 | 2.0 | 0.7 | 2.2 | 0.9 |
| 16 | 8.1 | 0.6 | 8.2 | 0.7 |
| 17 | 5.7 | 1.6 | 5.9 | 1.7 |
| 18 | 7.0 | 1.2 | 6.9 | 1.1 |
| 19 | 1.8 | 0.9 | 2.6 | 1.2 |
| 20 | 4.0 | 0.9 | 4.1 | 0.9 |
| 21 | 2.5 | 1.1 | 2.6 | 1.1 |
| 22 | 4.8 | 0.7 | 4.9 | 1.0 |
| 23 | 2.2 | 1.0 | 2.8 | 1.4 |
| 24 | 5.7 | 1.1 | 6.0 | 1.0 |
| 25 | 4.4 | 1.2 | 5.5 | 1.3 |

For peer review only

Table A3: Pearson correlation between same-day lunchtime pain scores and step count by participant ID

| Participant ID | Pearson correlation | 95% confidence interval |
|---|---|---|
| 1 | 0.05 | -0.25,0.35 |
| 2 | 0.35 | 0.054,0.58 |
| 3 | 0.00 | -0.37,0.37 |
| 4 | 0.17 | -0.07,0.39 |
| 5 | -0.21 | -0.5,0.11 |
| 6 | -0.62 | -0.95,0.38 |
| 7 | 0.27 | 0.047,0.46 |
| 8 | 0.09 | -0.15,0.32 |
| 9 | 0.11 | -0.24,0.43 |
| 10 | 0.02 | -0.26,0.3 |
| 11 | -0.01 | -0.27,0.25 |
| 12 | -0.12 | -0.33,0.094 |
| 13 | 0.18 | -0.061,0.39 |
| 14 | -0.05 | -0.34,0.25 |
| 15 | 0.23 | -0.013,0.44 |
| 16 | 0.06 | -0.36,0.45 |

For peer review only

| | | |
|---|---|---|
| 17 | 0.16 | -0.088,0.38 |
| 18 | -0.11 | -0.48,0.3 |
| 19 | -0.06 | -0.28,0.17 |
| 20 | 0.00 | -0.24,0.24 |
| 21 | -0.32 | -0.65,0.1 |
| 22 | 0.05 | -0.22,0.32 |
| 23 | 1.00* | NA |
| 24 | 0.00 | -0.2,0.19 |
| 25 | 0.25 | 0.0091,0.47 |

Footnote: *This was based on only two data points.

For peer review only

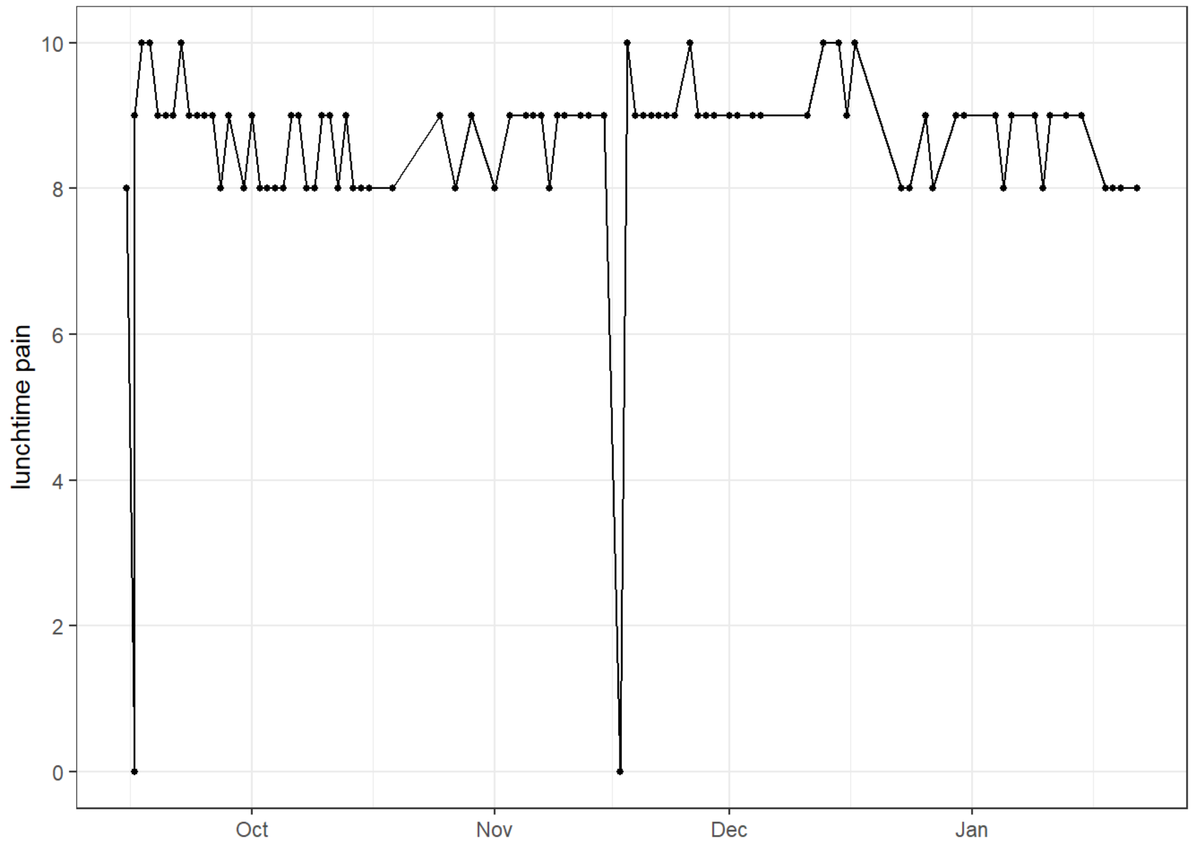


Figure A1: An example of a user with what we believe shows ‘false zeros’ (i.e., entered by mistake due to user interface error)

view only

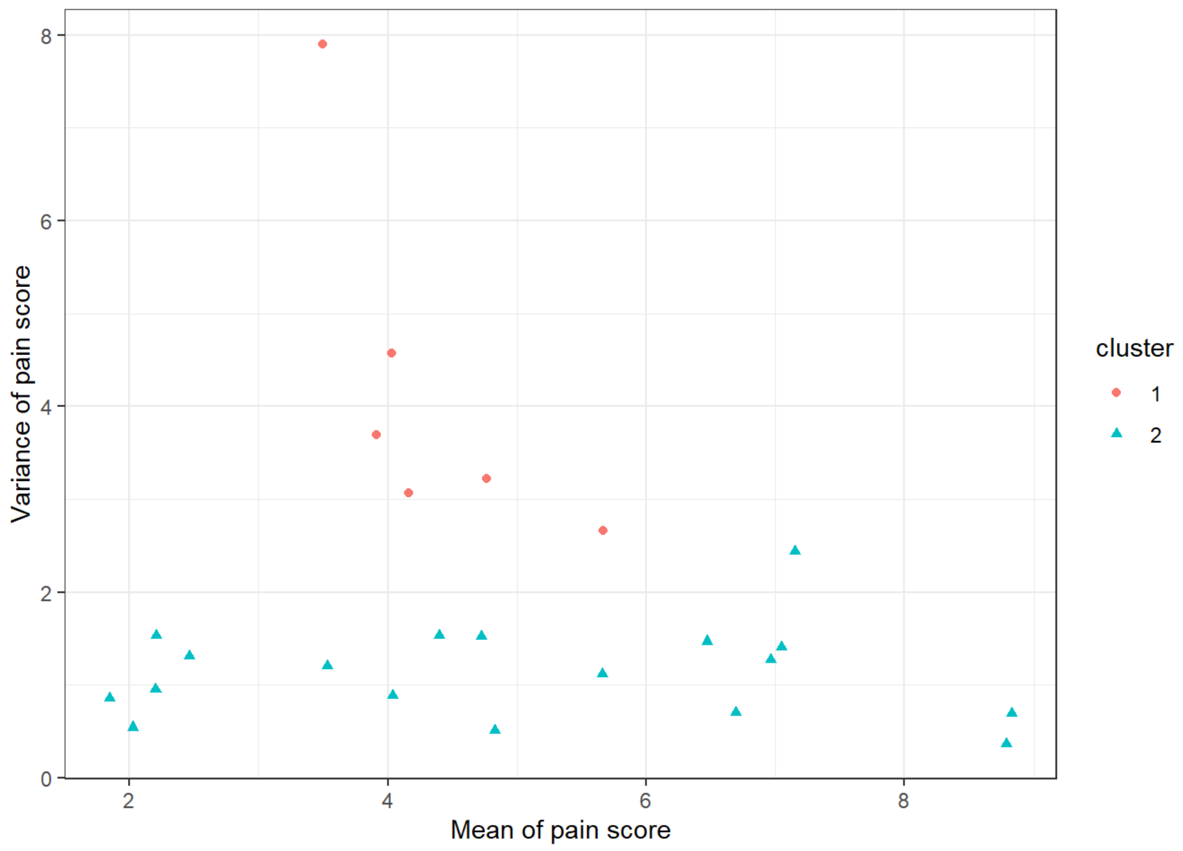


Figure A2: Two class model

For peer review only

**R code used for analysis**

````
```{r data, echo = TRUE}
pain <- read.csv('PainArani.csv')
koos <- read.csv('koosArani.csv')
steps <- read.csv('daily_steps.csv')
demographics <- readxl::read_excel('baseline.xlsx')
```
````

## Remove artifacts

For the pain dataset, we decided to only include those who had pain scores >0 (as we believed that scores of 0 may have been by accident).

Pain scores of zero were treated as missing values, due to the interface of the smartwatch app occasionally generating zero-values by accident as an error in data input.

Of `r nrow(pain)` total records, `r sum(pain$pain == 0, na.rm = TRUE)` had value zero and were removed.

This may include both 'true' zeros as well as those that were entered by mistake; we have no way to tell which might be which.

````
```{r artifacts}
pain <- subset(pain, pain > 0)
```
````

## Clusters

Cluster participants into three groups based on the means and variances of their morning and afternoon pain scores. We will use the *k*-means algorithm for this.

````
```{r clusters}
library(lubridate)
library(dplyr)
````

For peer review only
````

~~~
pain_summary <- pain %>%
  # Include 'overall morning' and 'overall pain' scores only.
  filter(grepl('MORNING|AFTERNOON', notification_type)) %>%
  group_by(user_id, notification_type) %>%
  # Compute summary statistics for each user.
  summarise(mean = mean(pain), var = var(pain)) %>%
  # Housekeeping
  rename(type = notification_type) %>%
  mutate(type = tolower(gsub('PAIN_OVERALL_', '', type)))

# Reshape the dataset.
pain_wide <- pain_summary %>%
  tidyr::pivot_wider(user_id, names_from = type, values_from = 
c(mean, var)) %>%
  na.omit() # remove user 37 who now has no data points for 
afternoon pain (after removing zeros)

knitr::kable(pain_wide %>%
               transmute(user_id,
                         mean_morning, sd_morning = 
sqrt(var_morning),
                         mean_afternoon, sd_afternoon = 
sqrt(var_afternoon)),
             digits = 1, caption = 'Means and standard deviations of 
pain scores by user')

# Apply the k-means algorithm to the means and variances.
# Run 10 times to avoid risk of local optima.
km <- kmeans(pain_wide[, -1], 3, nstart = 10)
clusters <- tibble(user_id = pain_wide$user_id, cluster = 
km$cluster)
```
~~~

We create a lookup table so it is easy to cross-reference the alpha-numeric Koalap IDs, the numeric user IDs and the cluster assignments.

~~~
```{r lookup}
~~~

For peer review only

```
user_lookup <- pain %>%
  distinct(user_id, KOALAP.Name) %>%
  left_join(clusters, by = 'user_id')
```

## Baseline demographics

To calculate BMI for each participant, convert to the appropriate units, then

use the formula

$$\text{BMI}_{\text{kg m}^{-2}} = \frac{\text{Weight}_\text{kg}}{(\text{Height}_{\text{m}})^2}.$$

```{r BMI}
demographics <- subset(demographics, `Koalap ID` != '23')
demographics <- within(demographics, {
  # Fix malformatted user ID:
  `Koalap ID` <- ifelse(`Koalap ID` == '29 & 5', 5, `Koalap ID`)
  Weight_kg <- as.numeric(gsub(' kg', '', Weight))
  feet <- as.numeric(substring(Height, 1, 1))
  inches <- as.numeric(gsub('[0-9] ft ?', '', Height))
  inches[is.na(inches)] <- 0
  Height_m <- 2.54 * (12 * feet + inches) / 100
  BMI <- Weight_kg / Height_m^2
})
```

## Knee injury and osteoarthritis outcome scores (Koos)

We need to convert the categorical Koos responses to numeric values and get total scores for each participant for each month.

```{r KOOS}
koos_scale <- c(None = 0, Mild = 1, Moderate = 2, Severe = 3,
Extreme = 4)

# Convert categorical responses to numeric values.
```

For peer review only

````
koos_num <- koos %>%
  mutate_at(vars(-(1:3)), function(x) koos_scale[x]) %>%
  mutate(timestamp = as_datetime(timestamp),
         month = round_date(timestamp, 'months')) %>%
  rename(KOALAP.Name = user_id) %>%
  left_join(user_lookup, by = 'KOALAP.Name') %>%
  # Some users answered questions multiple times. Take the latest response.
  group_by(user_id, month) %>% filter(row_number() == n()) %>%
  # We are only interested in questions P2 to P9.
  select(-KOOS_P1, -starts_with('KOOS_A'))

# Calculate monthly Koos for each participant, as a percentage.
total_possible_koos <- sum(grepl('KOOS', colnames(koos_num))) * 4 # = 32
koos_totals <- koos_num %>%
  tidyr::pivot_longer(starts_with('KOOS_'), names_to = 'question') %>%
  group_by(user_id, month) %>%
  summarise(koos_score = 100 * sum(value) / total_possible_koos)

# Check the resulting scores are valid.
if (any(koos_totals$koos_score > 100))
  stop('Koos percentage cannot be more than 100')
```
````

Compare Koos with daily pain trajectories.

````
```{r koos v pain, fig.width = 10, fig.height = 7}
koos_totals_numeric_ids <- koos_totals %>%
  ungroup() %>%
  left_join(user_lookup, by = 'user_id') %>%
  mutate(month = as.Date(month)) %>%
  filter(!is.na(user_id), user_id != 23)

pain %>%
````

For peer review only

```
  mutate(timestamp.Day = as_date(timestamp.Day)) %>%
  filter(notification_type.Number == 15, # morning pain only
         !is.na(user_id), user_id != 23) %>% # get rid of user whose
id is 'NA'
  ggplot() +
  aes(timestamp.Day, pain) +
  geom_line() +                  # daily pain scores
  geom_point(aes(month, koos_score / 10), data =
koos_totals_numeric_ids,
             colour = 'red', size = 2) +
  facet_wrap(~as.numeric(as.factor(user_id))) +
  scale_y_continuous(breaks = seq(0, 10, by = 5)) +
  labs(x = 'Date', y = 'Pain / KOOS',
       title = 'Monthly KOOS scores and daily pain scores',
       subtitle = 'Based on overall morning pain scores')

pain %>%
  mutate(timestamp.Day = as_date(timestamp.Day)) %>%
  filter(notification_type.Number == 14, # afternoon pain only
         !is.na(user_id), user_id != 23) %>% # get rid of user whose
id is 'NA'
  ggplot() +
  aes(timestamp.Day, pain) +
  geom_line() +                  # daily pain scores
  geom_point(aes(month, koos_score / 10), data =
koos_totals_numeric_ids,
             colour = 'red', size = 2) +
  facet_wrap(~as.numeric(as.factor(user_id))) +
  scale_y_continuous(breaks = seq(0, 10, by = 5)) +
  labs(x = 'Date', y = 'Pain / KOOS',
       title = 'Monthly KOOS scores and daily pain scores',
       subtitle = 'Based on overall afternoon pain scores')
```

## Step counts

For peer review only

Calculate the mean and variance of step counts per user, and link these data with the clustering assignments.

```
```{r steps}
steps_summary <- steps %>%
  rename(KOALAP.Name = user_id) %>%
  left_join(user_lookup, by = 'KOALAP.Name') %>%
  group_by(user_id = user_id) %>%
  summarise(mean = mean(steps), var = var(steps), sd = sqrt(var))
```
```

```
```{r step trajectories, fig.width = 10, fig.height = 5}
steps %>%
  rename(KOALAP.Name = user_id) %>%
  left_join(user_lookup, by = 'KOALAP.Name') %>%
  # Get rid of user 'NA' and user 23 who disappeared.
  filter(!is.na(user_id), user_id != 23) %>%
  ggplot() +
  aes(as.Date(date), steps, group = user_id) +
  geom_point(size = 1) +
  facet_wrap(~as.numeric(as.factor(user_id))) +
  scale_x_date(date_breaks = '1 month', date_labels = '%b') +
  scale_y_continuous(labels = function(x) ifelse(x == 0, '0',
scales::unit_format(1, scale = 1/1000, unit = 'k', sep = '')(x))) +
  labs(x = NULL, y = 'step count') #+
  #theme(strip.text = element_blank())
```
```

```
```{r}
steps_summary %>%
  left_join(user_lookup) %>%
  group_by(cluster) %>%
  summarise(mean = mean(mean),
            sd = sqrt(mean(var)),
            var = mean(var))
```

For peer review only
```

````
steps_summary %>%
  left_join(user_lookup2) %>%
  group_by(cluster) %>%
  summarise(mean = mean(mean),
            sd = sqrt(mean(var)),
            var = mean(var))
```


## Demographics by cluster

Let's plot step counts and body mass index (BMI).

```{r step counts by BMI}
steps %>%
  rename(KOALAP.Name = user_id) %>%
  left_join(user_lookup, by = 'KOALAP.Name') %>%
  # Summary statistics of step counts.
  group_by(user_id) %>%
  summarise(Q1 = quantile(steps, .25),
            Q3 = quantile(steps, .75),
            median = median(steps),
            min = min(steps), max = max(steps)) %>%
  # Housekeeping (match up the data types).
  mutate(user_id = as.character(user_id)) %>%
  # Join with BMI data.
  left_join(demographics, by = c(user_id = 'Koalap ID')) %>%
  filter(!is.na(user_id), user_id != '23') %>%
  ggplot() + aes(BMI, median) +
  geom_linerange(aes(ymin = min, ymax = max), alpha = .3) + # min to max
  geom_linerange(aes(ymin = Q1, ymax = Q3), linetype = 'solid') + # IQR
  geom_point() +
  theme_classic() +
  ylab('Step counts')
```
````
````

### BMI distribution

```{r BMI qq}
qqnorm(demographics$BMI, main = 'Normal quantile-quantile plot of BMI',
       col = c(M = 'steelblue', F = 'tomato2')[demographics$Gender],
       pch = c(M = 16, F = 17)[demographics$Gender])
qqline(demographics$BMI)
legend('bottomright',
       legend = c('M', 'F'),
       col = c('steelblue', 'tomato2'),
       pch = 16:17)
```

In tabular form.

```{r BMI table}
summary(demographics$BMI) %>%
  as.list %>%
  as_tibble %>%
  knitr::kable()
```

Standard deviation:

```{r}
sd(demographics$BMI)
```

### Age distribution

```{r age qq}
qqnorm(demographics$Age, main = 'Normal quantile-quantile plot of age',
       col = c(M = 'steelblue', F = 'tomato2')[demographics$Gender],
```

For peer review only

```
      pch = c(M = 16, F = 17)[demographics$Gender])
qqline(demographics$Age)
legend('bottomright', legend = c('M', 'F'),
       col = c('steelblue', 'tomato2'),
       pch = c(16, 17))
```
```

```{r}
summary(demographics$Age) %>%
  as.list %>%
  as_tibble %>%
  knitr::kable()
```

```{r}
sd(demographics$Age)
```

## Quality of life score

```{r qol}
pain %>%
  filter(notification_type == 'PAIN_QUALITY_OF_LIFE',
         !user_id %in% c(23, 37)) %>%
  group_by(user_id) %>%
  summarise(mean_qol = mean(pain),
            var_qol = var(pain)) %>%
  left_join(user_lookup, by = 'user_id') %>%
  group_by(cluster) %>%
  summarise(mean = mean(mean_qol),
            var = mean(var_qol, na.rm = TRUE)) %>%
  knitr::kable(col.names = c('Cluster',
                             'Mean(QoL score)',
                             'Variance(QoL score)'),
```

For peer review only
```

```
               digits = 2, caption = 'Average within-person mean and
average within-person variance (excluding participants who gave only
one QoL response)')
```

## Difference in morning and afternoon pain

```{r morning afternoon difference, fig.width = 8, fig.height = 8,
fig.cap = 'Jittered scatter plot of daily overall afternoon pain
against overall morning pain, by participant, with a line of $y =
x$'}
pain %>%
  filter(grepl('MORNING|AFTERNOON', notification_type),
         user_id != 23) %>%
  mutate(type = tolower(gsub('PAIN_OVERALL_', '',
notification_type)),
         date = as.Date(timestamp.Day)) %>%
  tidyr::pivot_wider(c(user_id, date), names_from = type,
values_from = pain) %>%
  tidyr::unnest() %>%
  ggplot() +
  aes(morning, afternoon) +
  geom_abline(intercept = 0, slope = 1) +
  geom_jitter(alpha = .5, width = .25, height = .25) +
  facet_wrap(~as.numeric(as.factor(user_id))) +
  scale_x_continuous('Morning pain', breaks = c(0, 5, 10)) +
  scale_y_continuous('Afternoon pain', breaks = c(0, 5, 10))
```

```{r morning afternoon timeline, fig.width = 8, fig.height = 8,
fig.cap = 'Difference between morning and afternoon overall pain
scores, over time, by participant'}
pain %>%
  filter(grepl('MORNING|AFTERNOON', notification_type),
         user_id != 23) %>%
  mutate(type = tolower(gsub('PAIN_OVERALL_', '',
notification_type)),
         date = as.Date(timestamp.Day)) %>%
```

````
  tidyr::pivot_wider(c(user_id, date), names_from = type,
values_from = pain) %>%
  tidyr::unnest() %>%
  ggplot() + aes(date, afternoon - morning) +
  geom_line() + geom_point() +
  facet_wrap(~ as.numeric(as.factor(user_id))) +
  xlab(NULL) +
  scale_y_continuous('(afternoon pain) - (morning pain)',
                     breaks = seq(-4, 4, by = 2), minor_breaks =
NULL)
```


```{r blandaltman, fig.width = 8, fig.height = 8, fig.caption =
'Tukey mean-difference plots (aka Bland--Altman plots) for morning
and afternoon pain'}
pain %>%
  filter(grepl('MORNING|AFTERNOON', notification_type),
         user_id != 23) %>%
  mutate(type = tolower(gsub('PAIN_OVERALL_', '',
notification_type)),
         date = as.Date(timestamp.Day)) %>%
  tidyr::pivot_wider(c(user_id, date), names_from = type,
values_from = pain) %>%
  tidyr::unnest() %>%
  mutate(diff = afternoon - morning,
         avg = (afternoon + morning) / 2) -> bland_altman


bland_altman_stats <- bland_altman %>%
  #filter(!is.na(diff)) %>% # weird bug where userid 42 (plot id 25)
gets omitted here?
  group_by(user_id) %>%
  summarise(mean = mean(diff, na.rm = TRUE),
            lower = mean(diff, na.rm = TRUE) - 1.96 * sd(diff, na.rm
= TRUE),
            upper = mean(diff, na.rm = TRUE) + 1.96 * sd(diff, na.rm
= TRUE))


ggplot(bland_altman) +
  aes(avg, diff) +
````

For peer review only

```
  facet_wrap(~ as.integer(as.factor(user_id))) +
  scale_x_continuous('average of lunchtime and early evening pain',
                     breaks = seq(0, 10, by = 2), minor_breaks =
NULL) +
  scale_y_continuous('(early evening pain) - (lunchtime pain)',
                     breaks = seq(-5, 5, by = 5), minor_breaks =
NULL) +
  geom_hline(aes(yintercept = lower), data = bland_altman_stats,
             linetype = 'dashed') +
  geom_hline(aes(yintercept = upper), data = bland_altman_stats,
             linetype = 'dashed') +
  geom_hline(aes(yintercept = mean), data = bland_altman_stats) +
  geom_point()
```

## Correlation between step count and pain

```{r correlation, fig.width = 8, fig.height = 8}
pain_and_steps <- pain %>%
  filter(notification_type == 'PAIN_OVERALL_MORNING', user_id != 23)
%>%
  transmute(user_id, date = as.Date(timestamp.Day), pain) %>%
  inner_join(steps %>%
               left_join(user_lookup, by = c(user_id =
'KOALAP.Name')) %>%
               transmute(user_id = user_id.y, steps, cluster, date =
as.Date(date)))

pain_and_steps %>%
  ggplot() + aes(pain, steps) +
  #geom_smooth(method = lm, se = FALSE) +
  geom_jitter(alpha = .5, width = .25, height = 0) +
  facet_wrap(~as.numeric(as.factor(user_id))) +
  scale_x_continuous('Lunchtime pain', breaks = c(0, 5, 10)) +
    scale_y_continuous('Steps', labels = function(x)
      ifelse(x == 0, '0', scales::unit_format(1, scale = 1/1000,
unit = 'k', sep = '')(x)))
```

For peer review only

~~~~
### Redo but for afternoon pain


pain_and_steps_pm <- pain %>%
  filter(notification_type == 'PAIN_OVERALL_AFTERNOON', user_id !=
23)  %>%
  transmute(user_id, date = as.Date(timestamp.Day), pain) %>%
  inner_join(steps %>%
               left_join(user_lookup, by = c(user_id =
'KOALAP.Name')) %>%
               transmute(user_id = user_id.y, steps, cluster, date =
as.Date(date)))


pain_and_steps_pm %>%
  ggplot() + aes(pain, steps) +
  #geom_smooth(method = lm, se = FALSE) +
  geom_jitter(alpha = .5, width = .25, height = 0) +
  facet_wrap(~as.numeric(as.factor(user_id))) +
  scale_x_continuous('Early evening pain', breaks = c(0, 5, 10)) +
    scale_y_continuous('Steps', labels = function(x)
      ifelse(x == 0, '0', scales::unit_format(1, scale = 1/1000,
unit = 'k', sep = '')(x)))
```
~~~~

If we wanted to compute a cohort-level correlation we could do so via the **rmcorr** package (we don't do that, here).

~~~~
```{r}
pain_and_steps %>%
  group_by(user_id) %>%
  summarise(step_pain_correlation = cor(pain, steps),
            conf_int = if (n() > 2) {
              paste(round(cor.test(pain, steps)$conf.int, 2),
collapse = ',')
            } else {
              NA_character_
~~~~

For peer review only

```
            }) %>%
  mutate(`ID on graphs` = as.numeric(as.factor(user_id))) %>%
  knitr::kable(col.names = c('Koalap ID', 'Correlation', 'Confidence
interval', 'User ID for graphs'),
               digits = 2, align = 'rrrr',
               caption = 'Correlation between same-day morning pain
score and step count, by user')


pain_and_steps_pm %>%
  group_by(user_id) %>%
  summarise(step_pain_correlation = cor(pain, steps),
            conf_int = if (n() > 2) {
              paste(round(cor.test(pain, steps)$conf.int, 2),
collapse = ',')
            } else {
              NA_character_
            }) %>%
  mutate(`ID on graphs` = as.numeric(as.factor(user_id))) %>%
  knitr::kable(col.names = c('Koalap ID', 'Correlation', 'Confidence
interval', 'User ID for graphs'),
               digits = 2, align = 'rrrr',
               caption = 'Correlation between same-day morning pain
score and step count, by user')
```


All of the analysis above excluded zeros from the analysis. Here we will redo it with zeros included, to highlight artifacts:

```{r raw scores, fig.width = 10, fig.height = 8}
pain0 <- read.csv('PainArani.csv')


pain0 %>%
  filter(grepl('MORNING', notification_type),
         user_id != 23) %>%
  mutate(date = as.Date(timestamp.Day)) %>%
  ggplot() + aes(date, pain) +
  geom_line() + geom_point(size = 1) +

For peer review only
```

```
  facet_wrap(~ as.numeric(as.factor(user_id))) +
  xlab(NULL) +
  scale_y_continuous('lunchtime pain',
                     breaks = seq(0, 10, by = 2), minor_breaks =
NULL)

pain0 %>%
  filter(grepl('AFTERNOON', notification_type),
         user_id != 23) %>%
  mutate(date = as.Date(timestamp.Day)) %>%
  ggplot() + aes(date, pain) +
  geom_line() + geom_point(size = 1) +
  facet_wrap(~ as.numeric(as.factor(user_id))) +
  xlab(NULL) +
  scale_y_continuous('early evening pain',
                     breaks = seq(0, 10, by = 2), minor_breaks =
NULL)

pain0 %>% count(user_id, pain) %>% filter(pain == 0)
```

```{r}
pain0 %>%
  filter(grepl('MORNING', notification_type),
         user_id == 18) %>%
  mutate(date = as.Date(timestamp.Day)) %>%
  ggplot() + aes(date, pain) +
  geom_line() + geom_point(size = 1) +
  xlab(NULL) +
  scale_y_continuous('lunchtime pain',
                     breaks = seq(0, 10, by = 2), minor_breaks =
NULL,
                     limits = c(0, 10))
```

For peer review only

STROBE Statement—checklist of items that should be included in reports of observational studies

| | Item No. | Recommendation | Page No. | Relevant text from manuscript |
|---|---|---|---|---|
| **Title and abstract** | 1 | (*a*) Indicate the study's design with a commonly used term in the title or the abstract | 1, 3, 4 | Observational, feasibility study. |
| | | (*b*) Provide in the abstract an informative and balanced summary of what was done and what was found | 3, 4 | Please see abstract. |
| **Introduction** | | | | |
| Background/rationale | 2 | Explain the scientific background and rationale for the investigation being reported | 6-7 | Please see 'introduction' section. |
| Objectives | 3 | State specific objectives, including any prespecified hypotheses | 7-8 | 1. To describe the day to day and within-day variability of pain intensity in people with symptomatic knee OA.<br>2. To assess how daily pain scores compare to pain assessed using monthly Knee Injury and Osteoarthritis Outcome Score (KOOS) pain domain scores.<br>3. To identify clusters of people with similar longitudinal pain scores.<br>4. To assess whether daily step count levels, assessed using the sensor data, are correlated with daily pain levels. |
| **Methods** | | | | |
| Study design | 4 | Present key elements of study design early in the paper | 8-9 | Please see 'study design and sample' section. |


For peer review only - http://bmjopen.bmj.com/site/about/guidelines.xhtml

| | | | | |
|---|---|---|---|---|
| Setting | 5 | Describe the setting, locations, and relevant dates, including periods of recruitment, exposure, follow-up, and data collection | 8-10 | Please see methods section. |
| Participants | 6 | (*a*) *Cohort study*—Give the eligibility criteria, and the sources and methods of selection of participants. Describe methods of follow-up<br>*Case-control study*—Give the eligibility criteria, and the sources and methods of case ascertainment and control selection. Give the rationale for the choice of cases and controls<br>*Cross-sectional study*—Give the eligibility criteria, and the sources and methods of selection of participants | 8 | Please see 'study design and sample' section. |
| | | (*b*) *Cohort study*—For matched studies, give matching criteria and number of exposed and unexposed<br>*Case-control study*—For matched studies, give matching criteria and the number of controls per case | N/A | N/A – not a matched study. |
| Variables | 7 | Clearly define all outcomes, exposures, predictors, potential confounders, and effect modifiers. Give diagnostic criteria, if applicable | 8-10 | Please see 'data collection' section. |
| Data sources/ measurement | 8* | For each variable of interest, give sources of data and details of methods of assessment (measurement). Describe comparability of assessment methods if there is more than one group | 8-10 | Please see 'data collection' section. |
| Bias | 9 | Describe any efforts to address potential sources of bias | 10-13 | Please see 'statistical methods' section. |
| Study size | 10 | Explain how the study size was arrived at | 8-9 | Please see 'sample size' section. |

Continued on next page

For peer review only

| | | | | |
|---|---|---|---|---|
| Quantitative variables | 11 | Explain how quantitative variables were handled in the analyses. If applicable, describe which groupings were chosen and why | 10-13 | Please see 'statistical methods' section. |
| Statistical methods | 12 | (*a*) Describe all statistical methods, including those used to control for confounding | 10-13 | Please see 'statistical methods' section. |
| | | (*b*) Describe any methods used to examine subgroups and interactions | 10-13 | Please see 'statistical methods' section. |
| | | (*c*) Explain how missing data were addressed | 13 | Please see 'missing data' section. |
| | | (*d*) *Cohort study*—If applicable, explain how loss to follow-up was addressed<br>*Case-control study*—If applicable, explain how matching of cases and controls was addressed<br>*Cross-sectional study*—If applicable, describe analytical methods taking account of sampling strategy | N/A | N/A |
| | | (*e*) Describe any sensitivity analyses | N/A | N/A |
| **Results** | | | | |
| Participants | 13* | (a) Report numbers of individuals at each stage of study—eg numbers potentially eligible, examined for eligibility, confirmed eligible, included in the study, completing follow-up, and analysed | 13 | Please see 'study characteristics' section. |
| | | (b) Give reasons for non-participation at each stage | 13 | Please see 'study characteristics' section. |
| | | (c) Consider use of a flow diagram | N/A | N/A |
| Descriptive data | 14* | (a) Give characteristics of study participants (eg demographic, clinical, social) and information on exposures and potential confounders | 13 | Please see 'study characteristics' section. |
| | | (b) Indicate number of participants with missing data for each variable of interest | N/A | N/A |
| | | (c) *Cohort study*—Summarise follow-up time (eg, average and total amount) | N/A | N/A |
| Outcome data | 15* | *Cohort study*—Report numbers of outcome events or summary measures over time | 14-16 | Outcomes reported under the relevant sub-headings. |
| | | *Case-control study*—Report numbers in each exposure category, or summary measures of exposure | N/A | N/A |
| | | *Cross-sectional study*—Report numbers of outcome events or summary measures | N/A | N/A |
| Main results | 16 | (*a*) Give unadjusted estimates and, if applicable, confounder-adjusted estimates and their precision (eg, 95% confidence interval). Make clear which confounders were adjusted for and why they were included | N/A | N/A |
| | | (*b*) Report category boundaries when continuous variables were categorized | 13-17 | Please see text for pain and step count variables. |


For peer review only - http://bmjopen.bmj.com/site/about/guidelines.xhtml

| | | | |
|---|---|---|---|
| | (*c*) If relevant, consider translating estimates of relative risk into absolute risk for a meaningful time period | N/A | N/A |

Continued on next page

For peer review only

| | | | | |
|---|---|---|---|---|
| Other analyses | 17 | Report other analyses done—eg analyses of subgroups and interactions, and sensitivity analyses | N/A | N/A |
| **Discussion** | | | | |
| Key results | 18 | Summarise key results with reference to study objectives | 17-20 | Please see 'discussion' section. |
| Limitations | 19 | Discuss limitations of the study, taking into account sources of potential bias or imprecision. Discuss both direction and magnitude of any potential bias | 19-20 | Limitations discussed. |
| Interpretation | 20 | Give a cautious overall interpretation of results considering objectives, limitations, multiplicity of analyses, results from similar studies, and other relevant evidence | 20-21 | Please see 'discussion' section. |
| Generalisability | 21 | Discuss the generalisability (external validity) of the study results | 20-21 | Please see 'discussion' section. |
| **Other information** | | | | |
| Funding | 22 | Give the source of funding and the role of the funders for the present study and, if applicable, for the original study on which the present article is based | 22 | Please see 'funding' sub-heading. |

*Give information separately for cases and controls in case-control studies and, if applicable, for exposed and unexposed groups in cohort and cross-sectional studies.

**Note:** An Explanation and Elaboration article discusses each checklist item and gives methodological background and published examples of transparent reporting. The STROBE checklist is best used in conjunction with this article (freely available on the Web sites of PLoS Medicine at http://www.plosmedicine.org/, Annals of Internal Medicine at http://www.annals.org/, and Epidemiology at http://www.epidem.com/). Information on the STROBE Initiative is available at www.strobe-statement.org.

For peer review only



For peer review only - http://bmjopen.bmj.com/site/about/guidelines.xhtml
